# Wetlands for wastewater treatment and subsequent recycling of treated effluent: a review

**DOI:** 10.1007/s11356-018-2629-3

**Published:** 2018-06-29

**Authors:** Suhad A. A. A. N. Almuktar, Suhail N. Abed, Miklas Scholz

**Affiliations:** 1 Civil Engineering Research Group, School of Computing, Science and Engineering, The University of Salford, Newton Building, Salford, England M5 4WT UK; 2 Department of Architectural Engineering, Faculty of Engineering, The University of Basrah, Al Basrah, Iraq; 3 Division of Water Resources Engineering, Department of Building and Environmental Technology, Faculty of Engineering, Lund University, P.O. Box 118, 221 00 Lund, Sweden; 4 Department of Civil Engineering Science, School of Civil Engineering and the Built Environment, University of Johannesburg, Kingsway Campus, Auckland Park, PO Box 524, Johannesburg, 2006 South Africa

**Keywords:** Constructed reed bed, Phytoremediation, Pollution control, Sustainable management, Treatment technology, Wastewater reclamation, Water reuse, Water scarcity

## Abstract

Due to water scarcity challenges around the world, it is essential to think about non-conventional water resources to address the increased demand in clean freshwater. Environmental and public health problems may result from insufficient provision of sanitation and wastewater disposal facilities. Because of this, wastewater treatment and recycling methods will be vital to provide sufficient freshwater in the coming decades, since water resources are limited and more than 70% of water are consumed for irrigation purposes. Therefore, the application of treated wastewater for agricultural irrigation has much potential, especially when incorporating the reuse of nutrients like nitrogen and phosphorous, which are essential for plant production. Among the current treatment technologies applied in urban wastewater reuse for irrigation, wetlands were concluded to be the one of the most suitable ones in terms of pollutant removal and have advantages due to both low maintenance costs and required energy. Wetland behavior and efficiency concerning wastewater treatment is mainly linked to macrophyte composition, substrate, hydrology, surface loading rate, influent feeding mode, microorganism availability, and temperature. Constructed wetlands are very effective in removing organics and suspended solids, whereas the removal of nitrogen is relatively low, but could be improved by using a combination of various types of constructed wetlands meeting the irrigation reuse standards. The removal of phosphorus is usually low, unless special media with high sorption capacity are used. Pathogen removal from wetland effluent to meet irrigation reuse standards is a challenge unless supplementary lagoons or hybrid wetland systems are used.

**Electronic supplementary material:**

The online version of this article (10.1007/s11356-018-2629-3) contains supplementary material, which is available to authorized users.

## Introduction and review purpose

### Background

Globally, the scarcity of freshwater is a growing problem and natural water resources are becoming inadequate to fulfill demand. This challenge is present worldwide, e.g., southern Europe, the Middle East, Australia, the southern states of the USA, and North Africa. According to Stikker (1998), the number of countries facing water scarcity during the last four decades, most of which are developing countries, is expected to increase to 34 by the year 2025 (Table 1). Gleick (1993) reported that about 80 countries around the world are expected to be suffering from serious shortages in water supply every year. According to Alcamo et al. (1997, 2000), 1.8 billion people are likely to face serious water scarcity challenges, and two thirds of the world may experience water shortage circumstances by 2025, while around half of the world will be under high water stress by 2030 (Scheierling et al. 2011). Moreover, the 2030 Water Resources Group (2030 WRG 2009) and World Water Assessment Programme (WWAP 2012) reported that the increase in water demand will be expected in all production sectors, and by 2030, 40% of the world will face water scarcity.

**Table 1:** Countries experiencing water scarcity in 1955, 1990, and 2025 (projected), based on availability of less than 1000 m^3^ of renewable water per person per year (refer to Stikker (1998) and UNESCO (2003) for more details)

Countries in water scarcity category			
In 1955	In 1990	By 2025, under all UN population growth projections	By 2025, only if they follow UN medium or high projections
Malta	Qatar	Libya	Cyprus
Djibouti	Saudi Arabia	Oman	Zimbabwe
Barbados	United Arab Emirates	Morocco	Tanzania
Singapore	Israel	Egypt	Peru
Bahrain	Tunisia	Comoros	Kenya
Kuwait	Cape Verde	South Africa	Algeria
Jordan	Kenya	Syria	
	Burundi	Iran	
	Algeria	Ethiopia	
	Rwanda	Haiti	
	Malawi	Somalia	
	Somalia	Malawi	
		Rwanda	

In addition to human population growth, the expansion of industrial and agricultural activities, global warming, and climate change are other reasons contributing to the water scarcity problems in many regions worldwide. However, the current situation in terms of water scarcity around the world is mostly because of both population and economic growth (Huang and Xia 2001). This is especially the case for low-income developing countries, which are categorized as poor in their unsatisfactory infrastructure for wastewater treatment (Varis and Somlyódy 1997).

As the population increases, the need for food and water will continually grow. As a result, actual water consumption will quickly approach the limits of the available resources leading to a reduction in productive agricultural area (FAO 2003). This will be the key reason for development limitation resulting in political, social, and economic challenge in such regions.

Population growth, which is considered as a demand pressure, will increase the urban, irrigation, and industrial water demand, which results in sharply rising discharges of various types of pollutants such as chemical and biochemical oxygen demands, particles (suspended solids and turbidity), ammonia-nitrogen, nitrate-nitrogen, hardly biodegradable organics (e.g., petroleum hydrocarbons, organic solvents, pesticides, and pharmaceuticals), heavy metals (e.g., cadmium, chromium, nickel, lead, copper, and zinc) and microbes (e.g., fecal coliforms and salmonella). These pollutants will cause a deterioration in the water quality of receiving watercourses, making these sources unsuitable for drinking, irrigation, and aquatic life.

Due to water scarcity problems around the world, it is essential to think about non-conventional water sources for fulfilling the increase in demand rate for freshwater. Wastewater is seen as a viable alternative option to overcome the shortage in water supply resulting from various reasons such as population growth (Bichai et al. 2012; Noori et al. 2014; Almuktar et al. 2015a, 2015b; Almuktar and Scholz 2015; Almuktar and Scholz 2016a, 2016b). However, the great variety in wastewater origins in terms of organic and inorganic constituents make the reuse of such water subject to regular monitoring to assess potential risks impacting on the total environment (FAO 2003). Adequate reuse of wastewater is essential to protect water resources, environment, and public health.

Direct disposal of untreated wastewater to land and water bodies has a negative impact on human health (Khurana and Pritpal 2012) and aquatic ecosystems (Scholz 2010). Because of this, wastewater treatment and recycling methods are vital to provide sufficient freshwater in the coming decades, since water resources are limited (FAO 2003). Wastewater remediation and reuse has been promoted due to an increase in the demand on water availability.

### Review purpose

Understanding the principles of urban wastewater reuse as an alternative and reliable source of water supply and analysis of the costs of wastewater reclamation are essential (Asano 1994; Mujeriego and Asano 1999). Therefore, this paper briefly reviews the global water scarcity challenge and focuses on treating wastewater using constructed wetlands, and subsequently reusing it for various purposes, but predominantly for irrigation saving freshwater resources for potable use. Wetland system characteristics, designs, and efficiencies in wastewater treatment for agricultural reuse are reviewed.

### Treated wastewater reuse opportunities

The treated wastewater effluent from municipal sewage systems is characterized as renewable, cheap, and attractive as a non-conventional water source. These pre-treated waters could be recycled for several reuse purposes including agriculture, aquifer recharge, industrial cooling, aquaculture, domestic applications (e.g., flushing of toilets), firefighting, parks and golf course watering, use of wetlands for wildlife habitats, and recreational impoundments (Asano et al. 2007) as highlighted below.

The potential reuse of wastewater depends on its characteristics, which determines the methods and degree of required treatment. Generally, agricultural irrigation reuse requires water treatment of low complexity. Minimum quality requirements for water reuse in agricultural irrigation have been developed (e.g., Alcalde Sanz and Gawlik 2017 and USEPA 2012) for key pollutants such as electric conductivity, total coliforms, and phosphorus (for more contaminants and corresponding thresholds, see Table 2). Rizzo et al. (2018) provides important comments on the European Union minimum quality requirements for water reuse in agricultural irrigation and aquifer recharge (Alcalde Sanz and Gawlik 2017).

**Table 2:** Irrigation water quality guidelines

Guideline	Unit	Westcot and Ayers (1985)	WHO (1989)	USEPA (2004)	Spanish Royal Decree ( 2007)	Italian Decree (2003)	FAO (1994, 2003) and Pescod (1992)
Irrigation parameter/type of guideline		Water quality for irrigation	Wastewater quality for agriculture	Reclaimed water quality for irrigation	Water quality for agriculture	Treated wastewater quality for reuse	Reclaimed water quality for irrigation
Salinity							
Electrical conductivity	dS/m	0.7–3^a^	–	–	3	–	0.7–3, 3^a^
Sodium adsorption ratio	–	–	–	–	6	10	0–15
Sodium	me/l	–	–	–	–	–	0–40
Magnesium	me/l	–	–	–	–	–	0–5
Calcium	me/l	–	–	–	–	–	0–20
Carbonate	me/l	–	–	–	–	–	0–0.1
Bicarbonate	me/l	–	–	–	–	–	0–10
Chloride	me/l	–	–	–	–	–	0–30
Sulfate	me/l	–	–	–	–	–	0–20
Total dissolved solids	mg/l	450–2000^a^	–	500–2000	–	–	450–2000^a^
Suspended solids	mg/l	–	–	–	20	10	–
pH	–	6.5–8	–	6	–	6–9.5	6.5–8.4
Pathogenicity							
Intestinal nematodes	eggs/l	–	< 1^c^	–	–	–	–
*Escherichia coli*	eggs/10 l	–	–	–	1^l^	–	–
	CFU/100 ml	–	–	–	100	100	–
Fecal coliforms	CFU/100 ml	–	< 1000^c^	–	–	–	–
Thermotolerant coliforms	CFU/100 ml	–	–	–	–	–	–
Total coliforms	CFU/100 ml	–	–	0–1000^d, e^	–	–	–
Nutrients							
Nitrate-nitrogen	mg/l	–	–	–	5.5	–	5–30^a^
Ammonia-nitrogen	mg/l	–	–	–	–	–	0–5
Total nitrogen	mg/l	–	–	10^d, f^	10	15	–
Phosphorus	mg/l	–	–	5^d, g^	–	2	0–2
Potassium	mg/l	–	–	–	–	–	0–2
Heavy metals and trace elements							
Aluminum	mg/l	–	–	5, 20^h^	–	1	5
Arsenic	mg/l	–	–	0.1, 2^h^	0.1	0.02	0.1
Beryllium	mg/l	–	–	0.1, 0.5^h^	0.1	0.1	0.1
Cadmium	mg/l	–	–	0.01, 0.05^h^	0.01	0.005	0.01
Cobalt	mg/l	–	–	0.05, 5^h^	0.05	0.05	0.05
Chromium	mg/l	–	–	0.1, 1^h^	0.1	0.005	0.1
Copper	mg/l	–	–	0.2, 5^h^	0.2	1	0.2
Iron	mg/l	–	–	5, 20^h^	–	2	5
Lithium	mg/l	–	–	2.5, 2.5^h^	–	–	2.5
Manganese	mg/l	–	–	0.2, 10^h^	0.2	0.2	0.2
Molybdenum	mg/l	–	–	0.01, 0.05^h^	0.01	–	0.01
Nickel	mg/l	–	–	0.2, 2^h^	0.2	0.2	0.2
Lead	mg/l	–	–	5, 10^h^	–	0.1	5
Selenium	mg/l	–	–	0.02, 0.02^h^	0.02	0.01	0.02
Vanadium	mg/l	–	–	0.1, 1^h^	0.1	0.1	0.1
Zinc	mg/l	–	–	2, 10^h^	–	0.5	2
Boron	mg/l	–	–	–	–	–	0.7–3^a^, 0–2

^a^For a slight to moderate degree of restriction on use

^b^For surface and sprinkler irrigation, respectively

^c^Irrigation of crops likely to be eaten uncooked, cereal crops, and industrial crops

^d^Food crops

^e^Value depends on the state of the USA, treatment degree of the water, and type of crop (raw, edible)

^f^Parameter only set for the state of New Jersey

^g^Parameter only set for the state of Michigan

^h^Long-term and short-term irrigation

^i^Sensitive, moderately sensitive, and tolerant crops, respectively

^j^Raw human food crops with and without direct contact with irrigation water, respectively

^k^Maximum concentration (mg/l) which can be tolerated for 20 and 100 years, respectively

^l^Crop irrigation using a system whereby reclaimed water comes into direct contact with edible parts of crops to be eaten raw

In contrast, domestic reuse options (direct or indirect potable and non-potable) demand high treatment. Treatment requirements for other reuse options lie between these two extremes (USEPA 2012; FAO 2012). Agricultural irrigation has, by far, been the largest reported reuse option of wastewater. In Japan, about 41% of recycled water; 60% in California, USA; and 15% in Tunisia were used for this purpose. Furthermore, in developing countries, land application has always been the main means of disposing of urban wastewater as well as meeting irrigation needs. In China, about 1.33 million hectares of agricultural land were irrigated with untreated or partially treated wastewaters from cities, while more than 70,000 hectares of cropland in Mexico City were irrigated with treated wastewater (FAO 2003). Irrigation has the advantage of “closing-the-loop” combination of waste disposal and water supply. Irrigation reuse is also more advantageous, because of the opportunity of reducing the purification levels and subsequently saving the treatment costs, with the role of soil and crops as biological treatment facilities (FAO 2012). Industrial reuse of treated wastewater represents the main reuse next only to irrigation in both developed and developing countries.

Reused wastewater is ideal for many industrial purposes, which do not require high-quality water. Based on industry type, reclaimed water can be utilized for cooling water make-up, boiler feed water, process water, etc. (USEPA 2012). Moreover, treated wastewater meeting strict quality criteria (Table 2) can be planned for reuse for many non-potable purposes. Non-potable reuse can reduce water consumption from other sources and decrease the wastewater flow rate (USEPA 2012). Indirect potable reuse of treated wastewater may unintentionally occur, when wastewater is disposed of into a water body that is utilized as a source for potable water supply. Here, treated wastewater, which meets the criteria for potable reuse (except for total dissolved solids), will be diluted with water from other sources to meet this criterion, and used for potable purposes (WHO 2006). Another planned indirect potable reuse can be through groundwater recharge of treated wastewater. On the other hand, adding treated wastewater directly into the normal drinking water distribution system refers to direct potable reuse (WHO 2006).

Considering that more than 70% of water around the world are consumed for irrigation purposes (UNESCO 2003; Pedrero et al. 2010), the application of treated wastewater for agricultural irrigation has great potential (Meda and Cornel 2010), especially when incorporating the reuse of nutrients like nitrogen and phosphorous, which are important for plant production (Norton-Brandão et al. 2013). Furthermore, the use of wastewater for irrigation purposes is another non-conventional water resource option, which is widely implemented in developing countries with low income and in arid rich countries due to the high stress on water resources (WB 2000; Smit et al. 2001; FAO 2003). The use of wastewater for agricultural purposes is considered as the most traditional application.

Furthermore, the appropriate management of recycling wastewater in the agricultural sector will reduce soil and plant contamination in addition to the moderation of the shortage in water resources (FAO 2003). Wastewater treatment and recycling in agriculture is a common practice in arid and semi-arid regions, which are suffering from severe shortages in freshwater resources, supporting renewable agriculture and food systems. Also, there is substantial attention on the long-term effects of reclaimed wastewater on crops intended to be consumed by humans (FAO 2003; Pedrero et al. 2010). Table 2 summarizes various example guidelines concerned with the quality of irrigation water. The published standards compare well to one another for most water quality variables. However, new standards are likely to be developed as the accuracy of scientific analytical equipment improves and more knowledge of new pollutants emerges.

### Technologies applied in wastewater treatment and reuse for irrigation

#### Background concerning various technologies

Traditional systems for wastewater treatment require intensive energy for mechanical components with high operational and investment costs. In most developing countries, current systems for wastewater treatment are failing to treat wastewater adequately, because of high costs in terms of operation and maintenance as well as the absence of know-how and lack of authority (Mustafa 2013). Moreover, some water resources are contaminated, because of the discharge of raw wastewater into water bodies resulting in the deterioration of water quality and contamination of freshwater sources, which adversely impacts on irrigation, recreation, and fish production (Kivaisi 2001). For some developing countries, pollution of water is the main risk to public health. Therefore, it is essential to protect the present water resources by reclaiming the wastewater produced by human activities and foster recycling to alleviate the shortage in freshwater resources.

Evolving a combination of strategies that provide high-quality water for supply, managing the water demand, and a decrease in long-term stresses on water resources is more crucial due to the increase in population growth. However, there are numerous factors, which may affect the strategy to be used for dealing with the scarcity of water in specific regions such as the topography, soil conditions, and availability of technical and financial support (Cosgrove and Rijsberman 2000). It is essential to adopt sustainable treatment technologies that can be sufficiently used to treat wastewater in the long term.

A combination of high-technology systems for treating wastewater appears inappropriate, since it is often economically infeasible. Hence, there is a great need to develop suitable, inexpensive, and rapid wastewater treatment and reuse techniques instead of traditional and costly treatment systems (Kumar et al. 2012).

Online Resource 1 shows the current technologies applied in urban wastewater reuse for irrigation. The focus is on pollutants such as salinity, pathogens, heavy metals, and nutrients (Norton-Brandão et al. 2013). Moreover, the advantages and disadvantages of these technologies are listed as well. Online Resource 1 shows that compared to conventional treatment systems, constructed wetlands seem to be the technology of the highest ability in terms of pollutant removal and have advantages in terms of low maintenance costs and required energy.

Furthermore, constructed wetlands have a high potential to be applied in developing countries (Kivaisi 2001). Constructed treatment wetlands involve physical, biological, and chemical processes, similarly to those occurring in natural wetlands. Constructed wetlands are applied to control pollution in the environment by treating various wastewaters such as urban, industrial, agricultural, animal, and mine effluents (Scholz 2010; Vymazal 2011b; Sani et al. 2013) as well as petroleum (Scholz 2010; Tang et al. 2010; Wallace et al. 2011; Al-Baldawi et al. 2014; Vymazal 2014) and municipal wastewaters successfully (Scholz 2010; Dong et al. 2011; Sani et al. 2013; Paing et al. 2015).

Constructed wetlands are characterized by biological activities that are higher than those occurring in conventional treatment systems, which convert various pollutants into non-toxic by-products in the wastewater. Constructed wetlands have also been used for secondary or even tertiary treatment and reuse of wastewater (Kadlec and Wallace 2008). More details on constructed wetland background are available in Online Resource 2.

The purification function of a constructed wetland system involves interconnections of various wetland plants, soils, and microbial organisms supporting the treatment of wastewater (Vymazal 2014). The performance of a wetland system in terms of wastewater treatment is mainly dependent on the nature, design, plant type, and microbial activity and local weather conditions (Vacca et al. 2005; Picek et al. 2007; Ström and Christensen 2007; Weishampel et al. 2009; Scholz 2010).

#### Case studies on constructed wetlands for treated wastewater reuse

Several studies were undertaken using wetland technology for wastewater treatment and subsequent recycling of the effluent for various purposes. For example, in Queensland (Australia), free water surface and subsurface flow artificial wetlands were used to treat different wastewater types such as municipal wastewater, household effluent, gold mine leachate, and agricultural runoff. The wetlands were used for polishing wastewater; reducing biochemical oxygen demand, nutrients, and particles; and disinfection of wastewater (Greenway and Simpson 1996). In this study, the treated wastewater was reused for different purposes such as golf course irrigation, river discharge, natural wetland discharge, ground water infiltration, and pasture irrigation. The Ingham Wetland belonged to one of these projects in Australia consisting of three *U*-shaped channels with dimensions of 110 m × 12 m × 500 mm and a design detention time of 12 days. The wetland was planted with five macrophyte species, which was used to polish wastewater effluent to an acceptable standard for creek discharge and to eliminate chlorine as a disinfection process. This wetland achieved BOD reduction results of 48, 52, and 8% for BOD, total nitrogen, and total phosphorous, respectively (Greenway and Simpson 1996).

Greenway and Simpson (1996) also undertook a study of the Townsville Wetland, which was a *U*-shaped channel of 60 m × 4 m × 400-mm dimensions and a detention time of 5 days with six species of macrophytes (two floating, two submerged, and three emergent ones). Their results showed that the Townsville Wetland produced high-quality effluent with 67, 44, 74, 65, 91, and 6% reduction for BOD, suspended solids, total nitrogen, ammonia-nitrogen, nitrate-nitrogen, and total phosphorous, respectively.

The Blackall Wetland was another project studied by Greenway and Simpson (1996) consisting of four linear channels (120 m × 7 m × 600 mm) that were planted with three macrophyte species of 4-day detention time. The findings showed average BOD reductions of 46% and suspended solid reductions of 68%. However, only 3% of total phosphorus were eliminated. The researchers (Greenway and Simpson 1996) indicated that the wetland projects showed that a very good standard of treatment was being achieved, making them highly effective in achieving their reuse objective.

A horizontal surface-flow constructed wetland treatment system situated in Karachi (NED University of Engineering and Technology) was used for treating wastewater containing domestic sewage and low flows from laboratories of various university departments aiming to assess the application of constructed wetlands for reuse (Mustafa 2013). The design of this pilot-scale constructed wetland consisted of a bed that is rectangular in shape with dimensions of 6-m height, 1.5-m length, and 0.6-m width; a surface area of 9 m^3^; a hydraulic detention time of 4 days; and a flow rate of 1 m^3^ per day planted with the common wetland plant *Phragmites karka* (Retz.) Trin. ex Steud. The system was monitored for 8 months for the period from September 2010 to April 2011. Results showed that the average reductions in BOD and COD were 50 and 44%, respectively. About 48% of effluent BOD concentrations were below the threshold of 30 mg/l. The suspended solid removal efficiency ranged from 73 to 86% with an average reduction of 78%. Roughly 38% of effluent SS concentrations were below the threshold of 30 mg/l. The average reduction in ammonia-nitrogen concentration for this study was 49%, while the average reduction in ortho-phosphate-phosphate concentration over the monitoring period was 52%. Moreover, the wetland reduced both total and fecal coliforms. The average removals of the analyzed indicator bacteria (total coliforms and fecal coliforms) were in the range from 93 to 99%, showing a high efficiency of the constructed wetland system in removing pathogens (Mustafa 2013).

Furthermore, Almuktar et al. (2017) assessed the possibility of recycling domestic wastewater treated by vertical-flow constructed wetlands for crop irrigation. The authors indicated that the studied wetlands showed high efficiencies in the removal of most contaminants meeting common standards of wastewater reused for irrigation shown in Table 2. In addition, wetlands were reported with the removal in the range of 55% for chromium (Cr) (Arroyo et al. 2010), between 25 and 35% for nickel (Ni), between 25 and 87% for zinc (Zn), about 9% for copper (Cu) (Galletti et al. 2010), 33% for cadmium (Cd), 75% for cobalt (Co) (Pedrero et al. 2010), and bacterial removal between 1 and 6 log units (Feigin et al. 2012) as shown in Online Resource 1, resulting in the consideration of wetland technology as the most attractive one for wastewater treatment and subsequent reuse (mainly for irrigation purposes). These studies indicated that if constructed wetlands are appropriately designed and operated, they could be used successfully for secondary and tertiary wastewater treatment under local conditions. Hence, constructed wetlands can be used in the treatment train to upgrade the existing malfunctioning wastewater treatment plants, especially in developing countries. The treated wastewater from these wetlands can be used for landscape irrigation and also for other beneficial uses (Mustafa 2013). According to Scholz (2010), the characteristics of the wastewater to be treated will decide the best wetland design (type) and properties to achieve the best treatment results meeting the required standards for reuse. The following sections discuss the constructed wetland types and classifications in greater detail.

#### Constructed wetland types and classifications

Generally, the classification of constructed wetlands is dependent on three main factors: water level in the system, which accordingly categorizes the constructed wetland as either free water surface flow or subsurface flow; macrophytes; and the direction of water movement in the system (Kadlec and Knight 1996; Langergraber et al. 2009; Nikolić et al. 2009; Hoffmann et al. 2011; Vymazal 2014). Moreover, constructed wetlands may also be classified according to their objectives into habitat creation, flood control, or wastewater purification, as reported in some recent studies (Vymazal 2013, 2014; Stefanakis et al. 2014).

However, Kadlec and Knight (1996), Kadlec et al. (2000), Langergraber et al. (2009), Knowles et al. (2011), Nivala et al. (2012), Vymazal (2013), and Wu et al. (2014) stated that surface flow and subsurface flow are considered as the main two flow types of constructed wetlands. The difference between these two types is that the first one includes substantial macrophytes and an exposed water surface while the second one has no clear water surface.

**Fig. 1:**
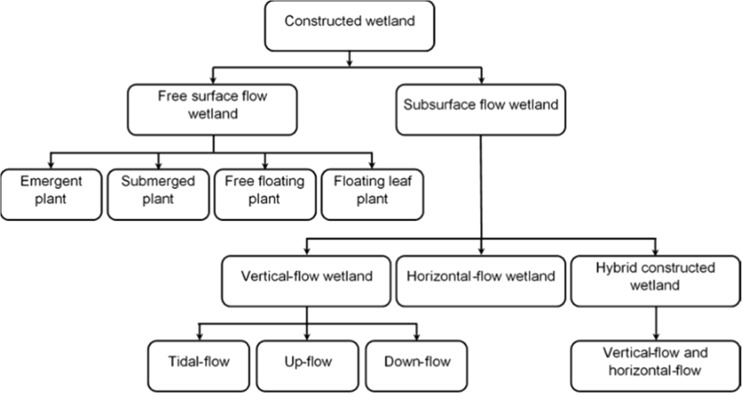
Constructed wetland classification

According to the direction of water movement in the system, constructed wetlands may be classified into vertical-flow and horizontal-flow types (Fig. 1), which can be combined into one single system (hybrid) to achieve a high pollutant removal efficiency (Vymazal 2013, 2014; Wu et al. 2014). Horizontal-flow constructed wetlands have substrate flooded by water, while vertical-flow constructed wetlands are ponded and drained with the intermittent application of water to the system (Stefanakis et al. 2014). The vertical-flow constructed wetland system was initially established and utilized by the German scientist Seidel in the early 1960s, as reported by Vymazal and Kröpfelová (2011). This type of wetland became popular for use after understanding the drawbacks of the horizontal systems in terms of nitrification incapability of the wastewater due to limitation of oxygen availability in such systems (Cooper 1999; Stefanakis et al. 2014).

Vertical-flow constructed wetlands achieve a high rate of oxygen transfer (Prochaska et al. 2007; Fan et al. 2013; Li et al. 2015). Initially, the applied wastewater in the wetland system will inundate the surface and then infiltrate through the system by gravity (Eke and Scholz 2008; Stefanakis et al. 2014). This will enhance the aeration and biological treatment in the system, when the air enters the pores as wastewater passes through the wetland media (Vymazal et al. 2006).

**Fig. 2:**
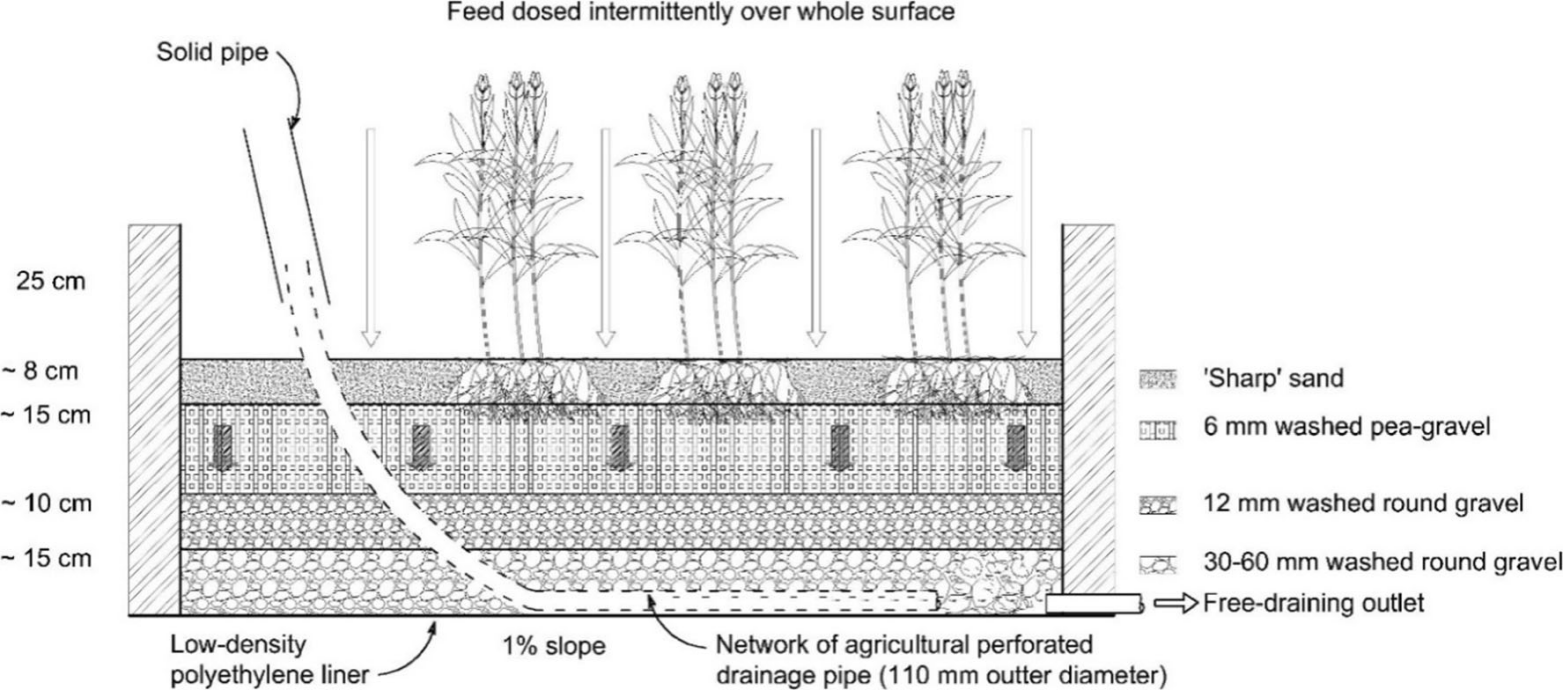
Typical arrangement of a vertical-flow constructed wetland allowing for a high outflow water per land area proportion, benefitting the agricultural sector

In vertical-flow constructed wetlands, the wastewater is applied intermittently (Fig. 2) in cycles of filling and draining the substrate media leading to a high rate of oxygen transfer in the system (Vymazal and Kröpfelová 2008; Wallace 2013; Li et al. 2015). This type of wetland has a low foot print allowing relatively high volumes of water to be treated per square meter, which is beneficial for the agricultural sector requiring high volumes of irrigation water. The applied wastewater floods the system and is then allowed to drain by gravity (Zhao et al. 2004). As a result, air enters the system pores and improves aeration and biological treatment (Vymazal et al. 2006; Fan et al. 2012; Song et al. 2015).

However, vertical-flow constructed wetlands are highly efficient in terms of treating different types of pollutants in the wastewater. For example, Prochaska et al. (2007) and Paing et al. (2015) indicated that vertical-flow constructed wetlands can remove chemical and biochemical oxygen demands as well as particles well from wastewater (Brix and Arias 2005; Scholz 2010). However, these systems are poor in terms of phosphorus removal due to insufficient interaction between wastewater and system media (Langergraber et al. 2007; Song et al. 2015). Moreover, many studies have shown that vertical-flow wetlands perform fine in terms of nitrification (Langergraber et al. 2007; Zhi et al. 2015), while others indicated their insufficiency in terms of denitrification (Scholz 2010; Vymazal and Kröpfelová 2011). However, denitrification in this system could be improved by a discontinuous loading regime amendment as discussed by Weedon (2003), Arias et al. (2005), and Weedon (2010).

In vertical-flow systems, substrate contains sand and/or gravel of a size distribution, which increases with depth (Vymazal et al. 2006). The substrate covers a depth of between 45 and 120 cm from top to bottom, and a slope ranging from 1 to 2% to enable treated wastewater to be drained and collected easily from the system outlet. Moreover, the discontinuous application of wastewater in vertical-flow constructed wetlands will provide the system with more oxygen due to air being sucked deep into the bed while draining the treated wastewater out of the system by gravity (Stefanakis et al. 2014). Moreover, this operation can be enhanced when aeration pipes are inserted in the system, leading to improvement in the nitrification processes and organic matter removal, if compared with the horizontal-flow constructed wetland system (Vymazal 2007; Kadlec and Wallace 2008; Stefanakis et al. 2014). The application of vertical-flow wetlands is more recently practiced in Africa and Asia (Kivaisi 2001; Abou-Elela et al. 2013; Wu et al. 2014). Biological or physical clogging in vertical-flow treatment wetlands is a problem, which affects their treatment efficiency. This could result from accumulation of biodegraded macrophytes, pollutants, and particles in the system leading to reduced pore volume, hydraulic conductivity, and permeability, which will greatly affect the quality of treated wastewater (Picard et al. 2005; Sani et al. 2013).

**Fig. 3:**
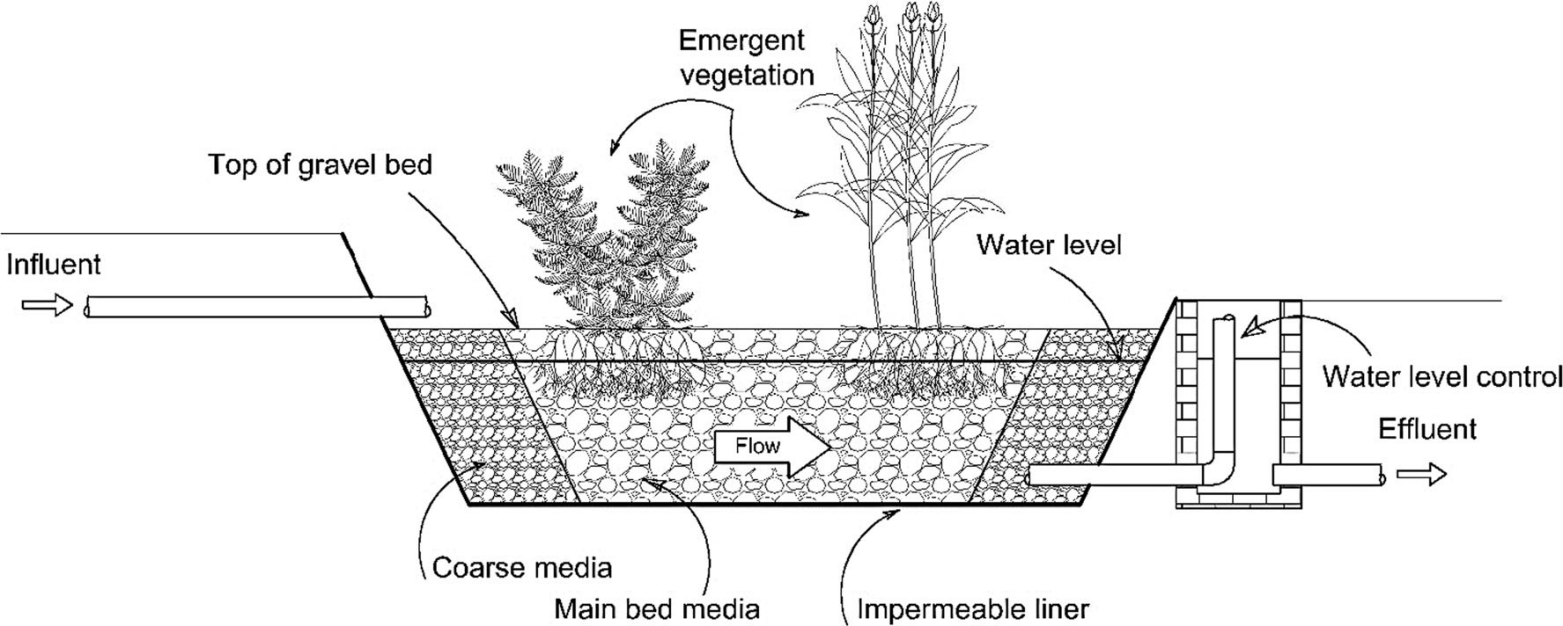
Schematic of a horizontal subsurface flow constructed wetland, which has high capital costs making it less attractive for the agricultural sector

**Fig. 4:**
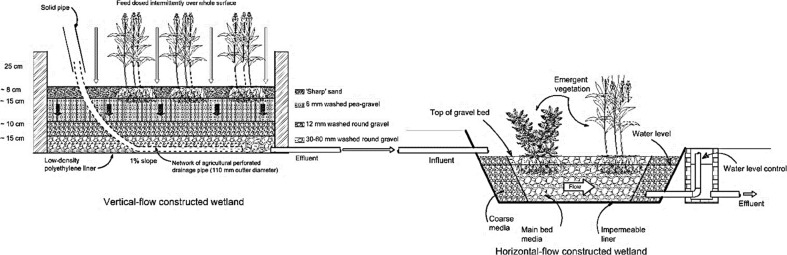
Hybrid constructed wetland arrangement

Another subsurface flow wetland type is the horizontal flow system in which wastewater moves horizontally through the system substrate, plant roots, and rhizomes toward the system outlet (Vymazal 2009, 2014). In this system, the treatment of wastewater, which floods the below-ground aggregates, is due to the interconnection of biological, chemical, and physical processes as wastewaters pass through aerobic, anaerobic, and anoxic zones (Kadlec and Knight 1996; Vymazal 2014). According to Brix (1987), the oxygen available in the aerobic system substrate is supplied by roots and rhizomes. Horizontal subsurface flow constructed wetlands are planted with macrophytes, which are established in the system substrate (Fig. 3) containing gravel and/or sand underneath, through which the applied wastewater passes from the system inlet toward the outlet (Vymazal et al. 2006). Typically, reeds (tall and grass-like wetland macrophytes) are used for horizontal subsurface flow constructed wetlands. In this system, the substrate depth ranges from 30 to 80 cm (Akratos and Tsihrintzis 2007) depending on the macrophyte types and their root depths with a slope between 1 and 3% supporting the gravitational flow of the applied wastewater. Moreover, the bottom of the system is sealed with an impermeable membrane avoiding leakage of the wastewater to the aquifer (Kadlec and Wallace 2008). Moreover, proper design of horizontal subsurface flow wetlands will allow the wastewater to be invisible at the surface of the system media and will enable it to remain about 5 to 15 cm below the surface (Vymazal et al. 2006). This will reduce the possibility of human exposure to pathogens and limit mosquito breeding (Kadlec and Wallace 2008). However, the roots of macrophytes and porous media in this system are responsible for biomass development and subsequently enhance organic matter and suspended solid removal from the contaminated water (Akratos and Tsihrintzis 2007; Gikas and Tsihrintzis 2010; Vymazal 2014). Compared with surface flow wetland systems, horizontal-flow constructed wetlands require a smaller land area, but incur high property investment costs as reported by Tsihrintzis et al. (2007), which makes them less attractive for the agricultural sector depending on cheap irrigation water. Moreover, horizontal subsurface flow constructed wetland systems have been applied in Europe and the USA (Vymazal 2014). Although horizontal subsurface flow constructed wetlands are reported to be poor in terms of ammonia-nitrogen removal, they can treat nitrate-nitrogen well due to the anoxic and anaerobic conditions available in horizontal subsurface flow constructed wetlands, which limit the nitrification of ammonia-nitrogen, but favor nitrate-nitrogen denitrification (Tunçsiper 2009; Zhang et al. 2014). In contrast, due to the availability of aerobic conditions in vertical subsurface flow constructed wetland systems, ammonia-nitrogen is removed well through nitrification processes, while nitrate-nitrogen is not, as denitrification is virtually absent in this system (Zhang et al. 2014). In other words, horizontal subsurface flow constructed wetlands are known to be good in denitrification, but poor in nitrification, while vertical subsurface flow constructed wetlands show contrary performances (Vymazal and Kröpfelová 2011; Vymazal 2014). This has led researchers to develop a combined wetland system consisting of both a horizontal subsurface flow constructed wetland together with a vertical subsurface flow constructed wetland (Fig. 4) aiming to obtain higher nitrogen removal (Vymazal 2005; Ayaz et al. 2012; Vymazal 2014).

The first hybrid constructed wetland system was developed in Germany between 1960 and 1969. A few similar systems were developed in France between 1980 and 1989, and then in the UK between 1990 and 1999 (Vymazal 2005). Currently, the use of this combined wetland systems is widespread around the world due to its efficiency in nitrogen compound removal from many wastewater types (Vymazal 2005; Vymazal and Kröpfelová 2011; Ayaz et al. 2012). Moreover, many studies have indicated that a hybrid wetland system could be used to treat different types of wastewater such as winery wastewaters (Serrano et al. 2011), pharmaceuticals (Reyes-Contreras et al. 2011), water produced from oil fields (Alley et al. 2013), grey water, and industrial wastewaters (Comino et al. 2013; Vymazal 2014).

**Fig. 5:**
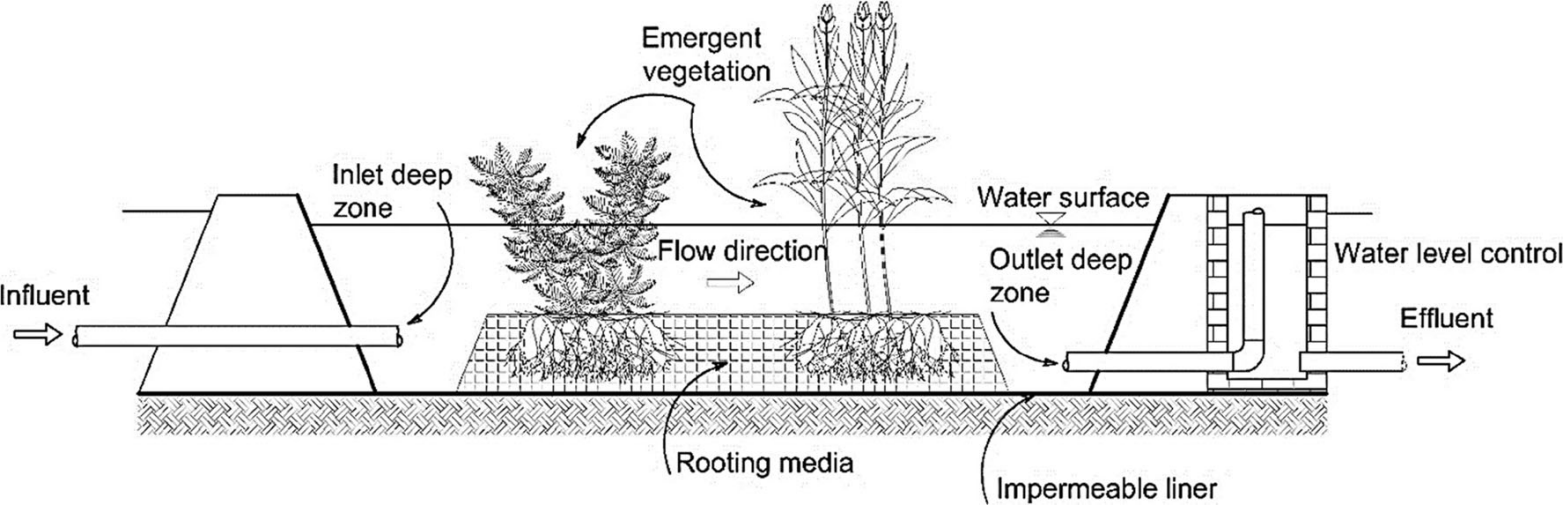
Free water surface flow constructed wetland configuration, which takes up a lot of potentially valuable farmland, making it an unattractive option for agricultural treated wastewater reuse

**Fig. 6:**
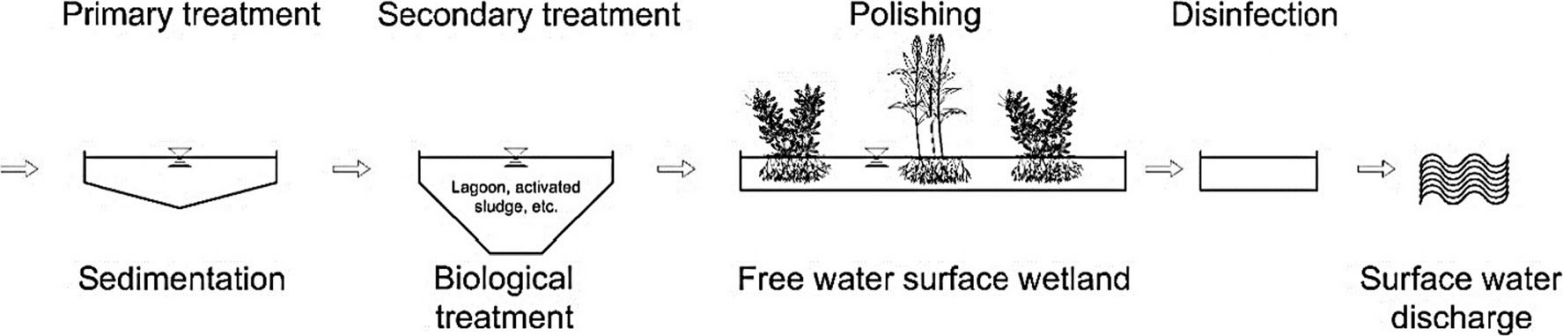
Typical application of a free water surface flow wetland for municipal wastewater treatment

Free water surface flow constructed wetlands are comprised of an exposed aquatic area covered with various plant types such as submersed, floating leaved, free floating, bottom rooted, or emergent macrophytes (Fig. 5). According to Vymazal et al. (1998, Vymazal et al. 2006) and Wu et al. (2014), the operation of free water surface constructed wetlands is similar to that of natural ones. This system consists of a sealed shallow pool to prevent wastewater leakage to the aquifer with a substrate of 40-cm-thick soil for establishing the macrophytes, as discussed by Stefanakis et al. (2014). In free water surface flow, the wastewater is loaded from the top; it then horizontally flows through the system media producing a water depth typically ranging from 20 to 40 cm, but a depth of up to 80 cm has also been reported by Vymazal et al. (2006) and Akratos et al. (2006). Moreover, treatment processes such as sedimentation, filtration, oxidation, adsorption, and precipitation will occur as wastewater passes through this wetland system (Kadlec and Wallace 2008). Since free water surface flow constructed wetlands closely simulate natural wetlands (Kadlec and Knight 1996), a high wildlife diversity is expected (insects, mollusks, birds, mammals, etc.). Moreover, these types of wetlands require a large land area, which make them unattractive for agricultural treated wastewater reuse and have a high potential for exposure of pathogens to humans (International Water Association (IWA) Specialist Group 2000). Because of the latter, free water surface flow constructed wetlands are infrequently used for wastewater treatment due to the high possibility of human exposure to pathogens (USEPA 2000). As a result, this type of wetland is usually applied for advanced effluent treatment from tertiary processes such as trickling filters, activated sludge systems, and lagoons (Fig. 6).

Free water surface flow constructed wetlands are suitable treatment technologies for the removal of suspended solids, nitrogen, heavy metals, biochemical oxygen demand, and pathogens (Vymazal 2007; Kadlec and Wallace 2008; Tsihrintzis and Gikas 2010). On the other hand, a subsurface flow constructed wetland systems consist of macrophytes planted on substrates of sand or gravel, allowing flooding of the system with wastewater, which will pass through the media by gravity, improving treatment processes (Knowles et al. 2011). The substrate arrangement in this system will provide an effective path that enhances the role of microorganisms in the system to treat various types of pollutants and allowing mechanisms such as adsorption and filtration to occur (Hoffmann et al. 2011).

Fan et al. (2012, 2013) and Nivala et al. (2013) reported that subsurface flow constructed treatment wetland systems show high efficiencies in terms of carbon and nitrogen compound removals, because of the elevated oxygen availability in their media. Moreover, this type of wetland shows good efficiency in small areas compared to those occupied by surface flow constructed wetlands as reported by Hoffmann et al. (2011) and Stefanakis et al. (2014).

Generally, a challenge concerning wetland performance is the limited removal efficiency of alkaline cations like sodium, calcium, and magnesium (Richardson 1989; Kohler et al. 2004; Samecka-Cymerman et al. 2004; Gu et al. 2006). This is because of the abundant availability of such cations in the wastewater, which commonly exceeds the plants’ needs, and subsequently their corresponding concentrations are unaffected when the wastewater passes through the wetland system (Richardson 1989). Samecka-Cymerman et al. (2004) observed that the removal efficiency of calcium and magnesium in wetland systems was higher during winter, when treatment is due to the activity of soil microbes rather than up-take by wetland macrophytes. Moreover, the up-take of cations in the tissue of wetland plants can be inhibited by elevated metal and hydrogen ion concentrations in the wetland system (Batty and Younger 2002). Evapotranspiration is another serious constraint, which limits recycling of wastewater treated by wetland systems due to the high rate of water losses (Green et al. 2006). Evapotranspiration can negatively impact on the potential reuse of (partially) treated wastewater for irrigation purposes by reducing the amount of irrigation water available for plants, which limits the surface area covered by irrigation water. Moreover, evapotranspiration in a wetland system will result in an increase of salinity and the concentration of contaminants in the effluent leading to the unsuitability of treated water for many crops (Coleman et al. 2001; Naylor et al. 2003; Xu and Jaffé 2006). However, evapotranspiration may have a positive effect due to the increase in concentration of some dissolved constituents in the rhizosphere of the wetland system resulting in increasing reaction rates, plant uptake, or both (Xu and Jaffé 2006) as well as in an improvement of the treated wastewater quality for irrigation reuse.

## Sustainable design and operation of constructed wetlands

### Constructed wetland vegetation

#### Background concerning vegetation

Macrophytes are common in wetlands (Vymazal 2002; Stefanakis et al. 2014), and are considered as a significant design element in natural and constructed systems (Scholz 2006, 2007, 2010; Villa et al. 2014). The presence or absence of these plants often defines wetlands (Saeed and Sun 2012) as green technology (Stefanakis et al. 2014). Macrophytes can absorb pollutants from the wastewater and accumulate them in their tissue in addition to providing microorganisms in the system with a complimentary growing environment as discussed by Vymazal (2002). Moreover, wetland macrophytes are responsible for transferring oxygen from their roots to the rhizosphere, providing aerobic conditions to enhance the contaminant degradation in the system (Moshiri 1993). This results in better wastewater treatment meeting the reuse standards for irrigation purposes (Marecos do Monte and Albuquerque 2010).

For example, in an intermittent loading system such as a vertical-flow constructed wetland, the macrophyte roots dissolve organic matter in wastewater, and subsequently prevent substrate from clogging by producing holes (after the degradation of dead rhizomes) for the water to pass through. Furthermore, growth of macrophytes in wetland substrate stabilizes media, which leads to the improvement of the hydraulic conductivity in the system, reduces clogging probability, and provides suitable conditions for microbial growth and release oxygen as reported by Li et al. (2008) and Stefanakis et al. (2014). The potentially key role of macrophytes and the impact of various other species of wetland plants on the significance of treatment efficiency for certain variables are disputed (Scholz 2006). However, other studies stated the substantial impact of macrophytes on wetland treatment systems in terms of contaminant removal. For example, Akratos and Tsihrintzis (2007) studied the reduction percentage in chemical and biochemical oxygen demand in planted wetlands and control systems. Their results showed that the mean reduction percentage in the planted wetlands (89%) was slightly greater than that of the controlled systems, which showed an average reduction percentage of 85%. Biochemical oxygen demand and total suspended solid reduction percentages (90 and 75%, respectively) were observed to be higher in the planted filter of a subsurface flow system compared to those in the controlled system, which showed reduction percentages of 46 and 63% in that order (Karathanasis et al. 2003). In Greece, a study was carried out to determine the reduction percentage of polycyclic aromatic hydrocarbons from urban wastewater using constructed wetlands and a gravel filter (Fountoulakis et al. 2009). The results indicated that the planted filter led to a reduction percentage of 79.0%, which was higher than that for the gravel filter of 73.3%. Furthermore, Paola and Elena (2014) indicated in their review paper that planted constructed wetlands generally remove pharmaceuticals from urban wastewater better than unplanted ones.

On the other hand, there are some studies, which have indicated that there is no substantial impact of wetland macrophytes in terms of pollutant removal in both planted and unplanted systems. For example, some researchers found that there was no difference in biochemical oxygen demand removal efficiency by constructed wetland systems during different times of plant growth (Scholz and Xu 2002; Scholz 2006), while other researchers found that there was no substantial difference in removal efficiencies in systems planted with different plant types like reeds, duckweed, and algae (Baldizon et al. 2002).

According to Kadlec and Knight (1996), a number of points should be considered when choosing wetland plants. For example, the chosen macrophytes should be sourced locally and have to be tolerant to waterlogged, anoxic, and hyper-eutrophic conditions. In addition, perennial plants, which live for more than 2 years or grow in two seasons, are preferable to enhance constructed wetland sustainability. Similarly, Wu et al. (2015) recommended that plants should be tolerant to hyper-eutrophic and waterlogged-anoxic conditions with a high capability for absorption of wastewater pollutants and a high climate change adaptation potential. Based on that and since the wetland vegetation has an important role in treatment processes as well as improvement of the effluent quality, this explain the vital role of this wetland element for treating the wastewater to be reused for various purposes, mainly those that do not require high-quality characteristics such as for irrigation reuse (Wu et al. 2016).

#### Macrophytes in constructed treatment wetlands

Wetland plants can be categorized under four main classes, namely, emergent plants, floating leave macrophytes, submerged plants, and freely floating macrophytes. Emergent macrophytes are known to stabilize substrate and are usually observed above the water surface. Moreover, these plants are grown in a water depth of around 50 cm above the soil (Saeed and Sun 2012; Vymazal 2011a). Macrophytes such as *Acorus calamus* L., *Carex rostrate* Stokes, *Phragmites australis* (Cav.) Trin. ex Steud., *Scirpus lacusris* (L.) Palla, and *Typha latifolia* L. (Saeed and Sun 2012) as well as genera such as *Iris* spp., *Juncus* spp., and *Eleocharis* spp. (Wu et al. 2015) are typical examples.

Floating leave plants are fixed in the saturated substrate. Typical water depths range from 0.5 to 3.0 m. Example species are *Nymphaea odorata* Aiton, *Nuphar lutea* (L.) Sm., *Nymphoides peltata* (S.G. Gmel.) Kuntze, *Trapa bispinosa* Roxb., and *Marsilea quadrifolia* L. plants (Saeed and Sun 2012; Wu et al. 2015).

Submerged macrophytes require aerated water for good growth. Moreover, the plant tissues responsible for photosynthetic processes are covered with water. However, these types of plants are mainly used to polish secondary treatment plants as stated by Saeed and Sun (2012). *Myriophyllum spicatum L*., *Ceratophyllum demersum L.*, *Hydrilla verticillata* (L.f.) Royle, *Vallisneria natans* (Lour.) H. Hara, and *Potamogeton crispus* L. are typical examples (Wu et al. 2015).

Freely floating plants drift on the water surface and have the ability to remove nitrogen and phosphorous from the wastewater through denitrification processes and subsequently combine them in their biomass. Moreover, these plants can remove suspended solids from wastewater (reducing the risk of clogging within sprinklers used for irrigation) as reported by Moshiri (1993). *Lemna minor* L., *Spirodela polyrhiza* (L.) Schleid., *Eichhornia crassipes* (Mart.) Solms, *Salvinia natans* (L.) All., and *Hydrocharis dubia* (Blume) Backer are characteristic examples, as indicated by Wu et al. (2015).

However, many studies have been undertaken to find the most popular plants used in wetlands worldwide. For instance, a survey on common emergent macrophytes used in free water surface flow constructed was undertaken by Vymazal (2013). His results showed that *P. australis* is the most popular plant in Europe and Asia, while *T. latifolia* was recorded as the most used species in North America. In Africa, *Cyperus papyrus* L. is commonly used, while *P. australis* and *Typha domingensis* Pers. as well as *Schoenoplectus tabernaemontani* (C. C. Gmel.) Palla are the most popular plants in Central and South Americas as well as Oceania, respectively.

Regarding the plant types used in subsurface wetlands, a review study undertaken by Vymazal (2011a) showed that *P. australis* is the most commonly used species globally. It is dominant particularly in Europe, Canada, Australia, Asia, and Africa.

Furthermore, *Typha* spp. such as *T. latifolia*, *T. domingensis*, *T. orientalis* C. Presl, and *T. glauca* Godr. are classified as the second most popular plants in subsurface flow wetlands found in Australia, North America, East Asia, and Africa. In addition, the *S. lacustris*, *S. californicus* (C.A. Mey.) Steud., *Eleocharis acuta* R.Br., and *S. tabernaemontani* are commonly used in New Zealand, North America, and Australia (Vymazal 2011a). However, *P. australis* is the most commonly used wetland plant for subsurface flow wetlands (IWA Specialist Group 2000; Scholz 2006; Vymazal 2014).

#### Macrophyte tolerance to wastewater to be used for subsequent irrigation

Plant tolerance is another crucial factor, which should be considered when choosing the specific plants for constructed wetlands as some plants may suffer from pollutants present in the wastewater resulting in limitation in both plant survival and treatment efficiency. This mainly occurs when applying a high load of wastewater or treating wastewater that contains abundant toxic contaminants (Moshiri 1993). Moreover, environmental stresses like eutrophication can damage wetland plants by inhibiting their growth or even causing their disappearance, with a direct effect on wetland treatment performance. According to Xu et al. (2010), excessive ammonia in wastewater can lead, for example, to physiological damage of plants and subsequent limitation of nutrient up-take by macrophytes.

However, visual symptoms linked to ammonia abundance can be observed as leave chlorosis, growth destruction, and root sinking as well as depression in plant yield (Xu et al. 2010). Based on this, several studies have been undertaken to evaluate the tolerance of wetland plants to different levels of contaminants available in wastewaters. For example, *T. latifolia* was reported to be stressed at ammonia concentrations ranging between 160 and 170 mg/l (Moshiri 1993), while *Schoenoplectus acutus* (Muhl. ex J. M. Bigelow) Á. Löve & D. Löve was noted as the only species among five types that was negatively affected by ammonia levels ranging between 20.5 and 82.4 mg/l during an experimental field study undertaken by Hill et al. (1997).

The physiological response of *P. australis* to different chemical oxygen demand concentrations was assessed by Xu et al. (2010). Their results showed that chemical oxygen demand concentrations of more than 200 mg/l can affect the plant metabolism processes, while concentrations exceeding 400 mg/l can result in obvious *P. australis* physiological changes. Also, *Arundo donax* L. and *Sarcocornia fruticose* (L.) A. J. Scott were reported to be very effective in removing high salinity, as well as organic matter, nitrogen, and phosphorus from wastewater (Calheiros et al. 2012), while *Typha angustafolia* L. was observed to remain alive at high chromium levels of 30 mg/l for a duration of 20 days, showing an outstanding accumulation ability (Chen et al. 2014). Moreover, *P. australis* was noted to tolerate and remove three antibiotics (ciprofloxacin, oxytetracycline, and sulfamethazine) available in wastewaters up to concentrations of 1000 μg/l (Liu et al. 2013). These studies are essential to understand the tolerance of different types of wetlands as well as to provide good information about the selection of the most tolerant species for treating wastewater using construction wetlands.

#### Pollutant removal capacity of macrophytes producing suitable irrigation water

Plants have an important role in wetland systems, which can directly affect the wastewater quality by improving various removal processes and consumption of phosphorous, nitrogen, and other elements (Ong et al. 2010; Ko et al. 2011). Moreover, antibiotics (Liu et al. 2013), nutrients (Scholz 2006, 2010; Vymazal 2007), and heavy metals (Scholz 2006, 2010; Ha et al. 2011) may accumulate in wetland plants. Several research studies have been undertaken to investigate the wetland plant uptake capacity. For example, Wu et al. (2013a, 2013b) performed a study on four emergent plant uptake capacities in a wetland system treating contaminated river water. The authors’ results reported nitrogen and phosphorous net uptake capacities of 6.50 to 26.57 g N/m^2^ and 0.27 to 1.48 g P/m^2^, respectively. However, the plant uptake capacity may differ for various reasons such as type of wastewater, hydraulic retention time, loading rate, weather conditions, and system arrangement as stated by Saeed and Sun (2012).

Furthermore, Greenway and Woolley (Greenaway and Woolley 2001) stated that wetland plants can remove a high percentage of nitrogen and phosphorous ranging from 15 to 80 and 24 to 80% for total nitrogen and total phosphorus, respectively, while Wu et al. (2013a, 2013b) found that these percentages only ranged between 14.29 and 51.89 and 10.76 and 34.17% for total nitrogen and total phosphorous removal in this order. With respect to the removal of heavy metals, Ha et al. (2011) studied the accumulation capacity of indium, lead, copper, cadmium, and zinc in *Eleocharis acicularis* (L.) Roem. & Schult. plants. Their results reported that these types of plants had an outstandingly positive ability to accumulate metals available in wastewater, making the outflow suitable for irrigation, if crops are sensitive to metals. However, Yadav et al. (2012) concluded that bioaccumulation of heavy metals depends not only on plant species but also on the specific part of the plant, as metals can be removed by the below-ground biomass more effectively than by the above-ground one.

There is a close relationship between nutrient content and increase in phytomass. The rapid increase in phytomass during the third and fourth months corresponded with high nutrient levels. Since plants store significant amounts of nutrient and trace elements during their growth, periodic harvesting of the above-ground plant parts is a recommended practice to remove significant amounts of nutrients (mainly during the first 5 months of growth) from the wastewater flowing into the wetlands. Wetland plant species with high phytomass productivity and a well-developed root system and ability to withstand flooding are most productive in nutrient removal. Plant harvesting in wetlands generally has a positive effect on nutrient removal such as TN, TP, COD, and BOD. Therefore, this method could be recommended as best wetland management practice to improve and maintain nutrient removal in constructed wetlands (Vymazal 2007).

### Constructed wetland substrate

Media used in constructed wetlands are named substrate or aggregate. Wetland media could be sand, gravel, rock, or organic material such as soil and compost, which provide the primary support for the wetland plants and microorganism growth, enhancing biodegradation of wastewater pollutants in addition to its impact on system hydrology mechanisms (Tietz et al. 2007; Meng et al. 2014). Moreover, wetland substrates remove contaminants from the wastewater by ion exchange, adsorption, precipitation, and complexation (Dordio and Carvalho 2013; Ge et al. 2015), enhancing the effluent quality to meet reuse standards in agriculture. However, the chemical composition of wetland substrate can affect the system efficiency. For example, soil of low nutrient content will lead to plants in the system to uptake nutrients from the applied wastewater directly improving the effluent quality and increasing the likelihood of meeting the standard for irrigation reuse (Wu et al. 2016).

Also, the gravel substrate in the system should be washed from time to time to enhance the filtration rate and reduce the clogging of system media. Furthermore, using a gravel substrate within a reed bed system will improve the nitrification process rate, while the use of soil media with such a system will increase the denitrification rate as discussed by Markantonatos et al. (1996). This will impact positively on plants to be irrigated with the treated wastewater due to disadvantages linked to ammonia abundance on crop growth and production (Almuktar et al. 2017). Moreover, substrate size and shape has an important role in the wetland system as it impacts on the surface area available for growing a biofilm and the system pore blockage probability.

Meng et al. (2014) reported that very large aggregate size will reduce the surface area available for microorganisms to grow, while Scholz and Xu (2002) indicated that small-sized media will support the growth of biofilms by increasing the available surface area supporting the microorganism community for better wastewater treatment biologically, resulting in better effluent quality for irrigation reuse (Wu et al. 2016). Furthermore, Hoffman et al. (Hoffmann et al. 2011) and Meng et al. (2014) concluded that the hydraulic loading rate in wetland systems, particularly subsurface flow types, can be directly affected by wetland aggregate porosity, as the clogging of wetland media is a common problem in such systems affecting the system performance, especially when using unsuitable media pores for the applied organic load.

The optimal selection of media depends on the purpose for which the wetlands have been designed for. Media size can vary from fine grain to field stone. Using coarse media within wetland systems will increase the hydraulic conductivity and lower the likelihood of system clogging, while fine media will remove suspended solids and turbidity well. This will improve the effluent quality supporting the reuse potential in agriculture (Wu et al. 2016). This is due to soil problems resulting from treated wastewater application for irrigation as wastewater particles may cause pore clogging of the soil affecting the aeration process of crop root system as well as the deterioration of soil permeability and other properties that subsequently affect negatively plants growth and productivity (Almuktar et al. 2017). For horizontal-flow constructed wetlands, the use of small grain size with low water depth will significantly improve the system performance and removal efficiency as reported by Laviranc and Mancini (Lavrinc and Mancini 2016). On the other hand, there might be a high potential for clogging to occur in such systems (Sundaravadivel and Vigneswaran 2001). More details on constructed wetland substrate are available in Online Resource 3.

Several studies have been undertaken to assess the impact of different substrates used to improve contaminant adsorption capacity. For example, Meng et al. (2014) confirmed the results obtained from previous studies (Saeed and Sun 2011; Tee et al. 2012; Saeed and Sun 2012), which assessed the use of different media substrates such as organic mulch and rice husk on system efficiency. The results showed that these substrates enhanced nitrogen removal due to organic carbon content. However, these results contradicted those of others regarding the use of expensive media to improve the wetland system performance. For instance, using granular activated carbon did not increase the adsorption capacity of constructed wetland media as shown by Scholz and Xu (2002). Moreover, using zeolite and bauxite substrates did not show a substantial enhancement in wetland system efficiency as reported by Stefanakis and Tsihrintzis (2012). Online Resource 4 displays the most common substrates used in constructed wetland systems. Considering that one of the most serious issues of irrigation with treated wastewater is the clogging of the irrigation system by effluent particles, which will also cause the clogging of the irrigated soils leading to infiltration and seepage problems, wetland substrate as well as the vegetation root systems will play a vital role in filtering the treated wastewater by trapping these particles during the treatment process (Wu et al. 2016; Lavrinc and Mancini 2016) resulting in better effluent properties for irrigation reuse (Almuktar et al. 2017).

### Constructed wetland microorganisms

Constructed wetlands considerably support microbial community growth, which plays a vital role in eliminating various types of wastewater pollutants during biological processes in addition to the physical processes (filtration and sedimentation), chemical transformations (reduction, oxidation, volatilization and precipitation), and the up-take by macrophytes in the constructed wetland system (Scholz 2006, 2010), which will enhance the quality of treated wastewater for irrigation reuse purposes.

According to Kadlec and Knight (1996), Paredes et al. (2007), Kadlec and Wallace (2008), and Shao et al. (2013), bacteria, fungi, algae, and protozoa can be considered as the main groups of microorganisms available in the aerobic and anaerobic zones of a wetland system. The important role of microorganisms in constructed wetlands is due to their microscopic size allowing contact with and feeding upon pollutants via their enzymes (Truu et al. 2009).

However, in the wetland system, biological, chemical, and physical process interactions result in organic pollutant treatment as well as phosphorous, nitrogen, and heavy metal transformations (Scholz 2006, 2010). For example, organics in the wetland system are removed by aerobic and anaerobic degradation processes, while nitrogen can be removed via microbial metabolism such as nitrification, ammonification, denitrification, and other processes (Meng et al. 2014).

Moreover, organic biodegradation is mostly linked to autotrophic bacteria, which produce organic compounds from inorganic carbon like carbon dioxide, and heterotrophic bacteria, fungi, and protozoa obtain their growth requirements from organic compounds (Kadlec and Wallace 2008). All fungi gain their growth requirement of nutrition and energy from organic matter (heterotrophic). More details on constructed wetland microorganisms are available in Online Resource 5.

Microorganisms in wetland systems can be highly active and dominant, if suitable conditions and adequate nutrients are available for growth and survival (Truu et al. 2009). According to Meng et al. (2014), the chemical biodegradation undertaken in a wetland system by microorganisms consists of complex biochemical processes, which differ according to the active microbial groups.

The role of wetlands in treating wastewater to be used for irrigation reuse purposes is considerably affected by microorganisms and their metabolism, media, and macrophyte roots, which can consume organic matter and nutrients, and subsequently reduce, break-down, or completely remove various pollutants from the treated wastewater to be reused in agriculture (Wetzel 1993; Faulwetter et al. 2009; Truu et al. 2009).

Microorganism groups in constructed wetland systems can be classified into internal and external microbes, which are characterized according to their activities (Truu et al. 2009). For example, the internal group, which lives in the system, is responsible for metabolic activity contributing to the treatment of pollutants, while pathogens in inflow wastewater, which are considered as external microbes, have no important impact on the wetland ecosystem, as they are unlikely to survive, since the ecosystem is antagonistic to external microbes (Vymazal 2005).

Wu et al. (2016) reported that the removal of such pathogens is a complex process that may be affected by operational factors such as the hydraulic regime, retention time, vegetation, seasonal fluctuation, and water composition. Moreover, the authors indicated that natural die-off due to starvation or predation, sedimentation, and filtration as well as adsorption are the most popular mechanisms for removal of these pathogens. Lavrinc and Mancini (2016) concluded that microbial parameters of constructed wetland effluent were the hardest to reach the irrigation reuse standards. Since the removal of these organisms is very important for human health protection, it is necessary to improve the wetland efficiency in that matter. For example, the authors reported that the storage of the effluent from wetlands in a lagoon proved beneficial for *Escherichia coli* removal. Also, they suggested that hybrid wetlands should be used to enhance the pathogen removal from the effluent as single-stage wetlands cannot meet the standards for irrigation reuse.

### Constructed wetland design and operational parameters

#### Key design and operational parameters

The continuous or discontinuous inundation of the wetland system substrate, which is linked to anaerobic conditions and provides a place where biogeochemical operations occur, is impacted upon by the local hydrology (Scholz 2010). In wetland systems, the hydro period and the depth of flooding are the main two parameters of wetland hydrology, which can directly affect nutrients, oxygen amounts, and pH as well as the wetland stability as discussed by Scholz (2006, 2010).

The time when the wetland media is water logged is defined as the hydro period, which can be affected by many features such as groundwater, geology, subsurface soil, topography, and climatic conditions. Moreover, the hydraulic retention time is defined as the average time for water to remain in the wetland. This time is a very crucial factor in wetland design and performance evaluation, mainly in the settling of solids, macrophyte uptake, and biochemical processes (Stefanakis et al. 2014). Several studies have been undertaken to monitor the impact of hydraulic retention time on treatment efficiency of a wetland system. For example, Akratos and Tsihrintzis (2007) studied the relationship between hydraulic retention time and chemical oxygen demand removal efficiency. The authors’ results show that with decreasing hydraulic retention time, the effluent chemical oxygen demand concentration will increase. These results were confirmed by Trang et al. (2010), who observed the reduction in organic matter and nitrogen removal efficiency with the reduction of hydraulic retention time in their system due to less contact time of contaminants in the wetland resulting in low effluent quality for reuse purposes in the agricultural sector. This drop in removal efficiency was observed in biochemical oxygen demand and total suspended solids as well as under short hydraulic retention times.

The effect of wetland design and operation parameters on the treatment efficiency of domestic wastewater was assessed by Dong et al. (2011). The authors’ reported that their wetland system showed high performance in removing contaminants. Their system achieved 98, 94, 92, 90, 96, 97, and 96% removal efficiency for biochemical oxygen demand, suspended solids, chemical oxygen demand, nitrate-nitrogen, total nitrogen, ammonia-nitrogen, and orthophosphate-phosphorus, respectively. However, Dong et al. (2011) concluded that these results were achieved because of the elevated hydraulic retention time of about 92 days.

The hydraulic retention time is one of the few operational factors, which can be controlled in wetland systems. For instance, a critical biochemical oxygen demand removal efficiency can be obtained at a hydraulic retention time of below 1 day, while the system efficiency will be enhanced at a hydraulic retention time of about 7 days as reported by Reed and Brown (1995). Based on this, hydraulic retention time is an important factor that affects the efficiency of the wetland system treatment, which is normally decided upon by designers. Despite the advantage of improving the treatment efficiency, when increasing the hydraulic retention time, this can also be considered as a main drawback for large wetland areas, particularly when land availability is restricted (Deblina and Brij 2010).

In wetlands, the surface loading rate is mainly dependent on the influent concentration and flow. However, the surface loading rate is difficult to control as the influent compositions vary significantly. An increase of influent flow will lead to an elevation in surface loading rate and decrease in hydraulic retention time (Scholz 2010). However, the wetland treatment efficiency depends on both hydraulic loading rate and hydraulic retention time as reported by Rousseau et al. (2008) and Abou-Elela et al. (2013). For example, in the case of a high hydraulic loading rate and a low hydraulic retention time, the pollutants in the wastewater will pass quickly through the wetland substrate without adequate contact time for biodegradation processes resulting in low treatment performance.

A low removal efficiency of a wetland system may be associated with fluctuations of the hydraulic loading rate, which is influenced by the hydraulic retention time and the applied loads, reducing the treatment capability of the bed (Marecos do Monte and Albuquerque 2010; Lavrinc and Mancini 2016). This can be explained by the slow development of the plants in the wetland system resulting in low removal in terms of nitrogen, total suspended solids, and biological and chemical loads (Lavrinc and Mancini 2016). Therefore, if the variation of the hydraulic loading rate could be controlled, the bed may reach a better performance, and a better quality of reclaimed water may subsequently be achieved for irrigation reuse (Marecos do Monte and Albuquerque 2010).

Other researchers have stated that ammonia-nitrogen can be removed well at long hydraulic retention times, regardless of the maturity of the wetland plants, while the chemical oxygen demand is unstable through experiments involving wetlands with mature macrophytes (Stefanakis and Tsihrintzis 2012; Zhi et al. 2015). However, a long resting time can also enhance the nitrification and biodegradation processes by supporting the system with artificial aeration time.

Furthermore, Tietz et al. (2007) and Stefanakis and Tsihrintzis (2012) indicated that organic matter breakdown mainly occurs in the top layers of a wetland system, predominantly in the upper layer (10–20 cm) due to the high availability of oxygen and microbial density in these layers. Flooding depth in a semi-natural wetland ranged between 2 and – 1 m (mean value of + 1 m) based on the ground surface (Scholz 2010).

#### Comparison of different wetland designs used for treated wastewater recycling

Table 3 summarizes specific design and operational recommendations for treating wastewater using constructed wetlands (Wu et al. 2015). However, more details on constructed wetland hydrology and surface loading rate are available in Table 4.

**Table 3:** Design and operation recommendations for treating wastewater using constructed wetlands (adapted from Wu et al. 2015)

Parameter	Design criteria	
	FWSF CW	SSF CW
Bed size (m^2^)	As larger as possible	< 2500
Length-to-width ratio	3:1–5:1	< 3:1
Water depth (m)	0.3–0.5	0.4–1.6
Hydraulic slope (%)	< 0.5	0.5–1
Hydraulic loading rate (m/day)	< 0.1	< 0.5
Hydraulic retention time (day)	5–30	2–5
Media	Natural media and industrial by-product preferred, porosity of 30 to 50%, particle size < 20 mm, 50–200 mm for the inflow and outflow	
Vegetation	Native species preferred, plant density 80% coverage	

*FWSF CW* free water surface flow constructed wetland, *SSF CW* subsurface flow constructed wetland

**Table 4:** Overview of constructed wetland design and operational parameters

Location	Wastewater (WW) type	Wetland design and operation				
		Plant	Dimension (*L* × *W* × *D*) (m × m × m)	Hydraulic loading rate, HLR (m^3^/day)	Hydraulic retention time, HRT (day)	Reference
Free water surface flow constructed wetlands						
Peradeniya, Sri Lanka	Municipal WW	*Scirpus grossus* L.f. *Typha angustifolia* L.	25.0 × 1.0 × 0.6	13	18 h	Jinadasa et al. (2006)
Nyanza, Kenya	Sugar factory WW	*Cyperus papyrus* L. *Echinochloa pyramidalis* (Lam.) Hitchc. & Chase.	3.0 × 20.0 × 0.4	75 mm/day	–	Bojcevska and Tonderski (2007)
Taihu, China	Lake water	*T. angustifolia*	20.0 × 1.5 × 0.8	0.64 m/day	–	Li et al. (2008)
Putrajayacity, Malaysia	Storm water	*Phragmites karka* (Retz.) Trin. ex Steud. *Lepironia articulata* (Retz.) Domin	1.5 × 0.7 × 0.8	0.17–0.63	–	Sim et al. (2008)
Shanghai, China	River water	*Phragmites australis* (Cav.) Trin. ex Steud.	800 m^2^ × 0.75 m	1,800	10	Li et al. (2009a); Li et al. (2009b)
EI, Salvador	Municipal WW	*T. angustifolia*	48.9 × 15.0 × 0.6	151.4	9.8	Katsenovich et al. (2009)
Liaohe, China	Oil-produced WW	*P. australis*	75.0 × 7.5 × 0.25	18.75, 37.5	15, 7.5	Ji et al. (2007)
Petchaburi, Thailand	Municipal WW/	*T. angustifolia*	4.0 × 1.0 × 1.5	6–150 mm/day	2; 5	Klomjek and Nitisoravut (2005)
Subsurface horizontal flow constructed wetlands						
Egypt	Greywater	*P. australis*	1.1 × 1.0 × 0.4	–	5	Abdel-Shafy et al. (2009)
	Blackwater	*P. australis*	1.1 × 1.0 × 0.4	–	10	
Juja, Nairobicity, Kenya	Municipal WW	*C. papyrus*	7.5 × 3.0 × 0.6	–	–	Mburu et al. (2012)
	Municipal WW	*C. papyrus*	7.5 × 3.0 × 0.6	–	–	
Dares Salaam, Tanzania	Municipal sludge	*Typha latifolia* L.	4.2 × 1.4 × 0.6	0.683	2.5	Kaseva (2004)
	Municipal sludge	*Phragmites mauritianus* Kunth.	4.2 × 1.4 × 0.6	0.683	2.5	
Dongying, Shangong, China	Municipal WW	–	35.2 ha × 0.5	50,000	1.8	Wang et al. (2006)
	Industrial WW	–	35.2 ha × 0.5	50,000	1.8	
Mother Dairy Pilot Plant, India	Municipal sludge	*P. australis*	69 × 46 × 0.3	43.05 l/m day	5.15	Ahmed et al. (2008)
Shatian, Shenzhen, China	Municipal WW	*Canna indica* L.	80 × 30 × 1.5	–	11.5	Shi and Wang (2004)
	Municipal WW	*Thaliade albata* Fraser ex Roscoe	58 × 20 × 1.6	–	8	
Dhaka, Bangladesh	Tannery WW	*P. australis*	1.3 × 1.0 × 0.8	6 cm/day	4.8	Saeed et al. (2012)
	Tannery WW	*P. australis*	1.3 × 1.0 × 0.8	6 cm/day	12.5	
Taihu, Zhejing, China	Lake water	*T. angustifolia*	20.0 × 1.5 × 1.0	0.64 m/day	–	Li et al. (2008)
Peradeniya, Sri Lanka	Municipal WW	*S. grossus*	1 × 25 × 0.6	–	18	Tanaka et al. (2006)
	Municipal WW	*Hydrilla verticillata* (L.f.) Royle	1 × 25 × 0.6	–	18	
Futian, Shenzhen, China	Municipal WW	*Kandelia candel* (L.) Druce	2 × 1 × 0.75	–	1, 2, 3	Yang et al. (2008)
	Municipal WW	*Aegiceras corniculatum* (L.) Blanco	2 × 1 × 0.75	–	1, 2, 3	
Wuhan, China	Municipal WW	–	3.0 × 0.7 × 1.0	130 l/day	–	Zhang et al. (2012)
EI, Salvador	Municipal WW	*P. australis*	18.3 × 7.3 × 0.6	151.4	–	Katsenovich et al. (2009)
Can Tho University, Vietnam	Municipal WW	*Phragmites vallatoria* Pluk. ex L.	12 × 1.6 × 1.1	31 mm/day	–	Trang et al. (2010)
				62 mm/day	–	
				104 mm/day	–	
				146 mm/day	–	
Subsurface vertical flow constructed wetlands						
Beijing, China	Municipal WW	*Salix babylonica* L.	1.5 × 0.8 × 1.0	0.12 m/day	–	Wu et al. (2014)
Shanghai, China	Municipal WW	–	–	0.76 m^3^/m^2^ day:0.04 m^3^/m^2^ day	–	Wang et al. (2006)
Kampala, Uganda	Municipal WW	*C. papyrus*	0.58 m^2^ × 0.82 m	0.064	5	Kyambadde et al. (2004)
Wuxi, China	Livestock WW	*P. australis*	2.0 × 2.0 × 1.0	0.4	–	He et al. (2006)
	Livestock WW	*Phragmites* spp. *Typha* spp.	2.0 × 2.0 × 1.0	0.4	–	
Guangzhou, China	Municipal WW	*Cyperus alternifoliu* var. gracilis	5.0 × 3.0 × 1.8	0.45 m^3^/m^2^ day	18	Chan et al. (2008)
Chiang Mai, Thailand	UASB effluent	*Scirpus grossus* L.f.	2.0 × 2.0 × 1.4	3, 6, 12 cm/day	–	Kantawanichkul et al. (2003)
Wuhan, China	Municipal WW	*Typha orientalis* C. Presl	1.0 × 1.0 × 1.0	250 mm/day	1.2	Chang et al. (2012)
	Municipal WW	*Canna indica* L.	1.0 ×1.0 × 1.0	250 mm/day	1.2	
Subsurface hybrid constructed wetlands						
Yongding River, China	Lake water	–	7.3 h m^2^	0.58 m^3^/m^2^ day	34.26 h	Liu et al. (2007)
Texcoco, Mexico	Municipal WW	*P. australis*	8.8 × 1.8 × 0.6	2.88	2.3	Belmont et al. (2004)
Nepal	Municipal WW	*P. karka*	8.0 × 9.5 × 0.5	0.13 m day	–	Singh et al. (2009)
	Municipal WW	*Canna latifolia* (Herb Smith)	10.0 × 7.5 × 0.6	0.13 m day	–	
Turkey	Municipal WW	*Iris hartwegii* subsp. *australis* (Parish) L.W. Lenz	1.5 × 3.5 × 0.4	60 l/ m^2^ day	–	Tunçsiper (2009)
	Municipal WW	*P. australis*	1.5 × 3.5 × 0.32	60 l/m^2^ day	–	
Ningbo, China	Municipal WW	*Taxodium ascendens* Brongn.	8 × 6 × 1	16 cm/day	5.4	Ye et al. (2001)
	Municipal WW	*Zizania aquatic* L.	7 × 5 × 3	32 cm/day	2.7	
Bogotá, Savannah, Columbia	Municipal WW	–	4,354 m^2^ × 0.6 m	40 cm/day	0.6	Arias and Brown (2009)
	Municipal WW	–	17,416 m^2^ × 0.5 m	10 cm/day	4.5	
Jakarta, Indonesia	Laboratory WW	*Typha* spp.	3.0 m^2^ × 0.4 m	250 l/day	1	Meutia (2001)
	Laboratory WW	*Lemna* spp.	3.0 m^2^ × 0.4 m	250 l/day	1	
Koh Phi, Thailand	Municipal WW	*Canna* spp., *Heliconia* spp. and	2,300 m^2^ × 0.7 m	400	–	Brix et al. (2011)
	Municipal WW	*Papyrus* spp*.*	750 m^2^ × 0.6 m	400	–	

*UASB* upflow anaerobic sludge blanket

The impact of water depth on treatment efficiency has been investigated by several authors. For example, Aguirre et al. (2005) studied the impact of flooding depth on efficiency of organic matter removal by using two subsurface horizontal flow constructed wetlands of different water depths (0.27 and 0.5 m). Their results showed that the shallow system gave better performance than the deep one, mainly in terms of biochemical oxygen demand, which showed removal efficiencies of 72 to 85% in shallow wetlands, and 51 to 57% in the deep ones, suggesting that metabolism pathways may differ with varying water depth.

The same observation was reported regarding pathogen removal in horizontal subsurface flow treatment wetlands, which showed better elimination of total coliforms and *E. coli* in shallow systems (Morató et al. 2014). Contrary to this, greater water depth is suggested to increase the contact time resulting in improving the treatment efficiency (Kadlec and Wallace 2008). However, the actual water depth in a wetland system is mainly dependent on the maximum depth of plant roots, which in turn is dependent on the selected wetland system plant types. As a result, the selected plant types will determine the substrate depth in the wetland bed, which should not be very deep; otherwise, the plant roots will not reach the system bottom leading to anaerobic conditions in this zone, which is devoid of roots (Scholz 2010). Furthermore, the water depth in the wetland is directly linked to the availability of oxygen in the system as the upper layers will be aerated by atmospheric diffusion while inside the system, and diffused oxygen from the plant roots will contribute to aeration. This means that the bottom layers of the system, which are not reached by roots, will lack oxygen resulting in anoxic or anaerobic conditions in these zones.

Table 4 provides an overview of constructed wetland design and operational parameters in developing countries. The information is not listed in any particular order.

### Influent feeding mode of constructed wetlands

The influent feeding mode is another crucial design factor that can affect the performance of a wetland system (Zhang et al. 2012). Wetlands can be fed in continuous, batch, and intermittent modes. These modes affect the oxidation and reduction conditions as well as the oxygen to be transferred and diffused in the system resulting in treatment efficiency modification. Accordingly, several studies have been performed to investigate the impact of feeding mode on wetland system treatment efficiency.

Wu et al. (2015) stated that the batch feeding mode generally showed the best performance compared to the continuous one as the former can provide more oxygen in the treatment system. These results were confirmed by Zhang et al. (2012), who performed a study to compare the removal efficiency in tropical subsurface flow treatment wetlands operated using batch and continuous modes. Their results showed that ammonia-nitrogen was removed with an efficiency of 95.2% in the batch mode system, which was significantly (*p* < 0.05) higher than that obtained from the continuous mode of 80.4% removal efficiency. Moreover, feeding the system intermittently can improve the removal of nitrogen and organic matter as reported by Saeed and Sun (2012).

For subsurface flow constructed wetlands, intermittent feeding systems show noticeable improvements in ammonium removal efficiency compared to continuous ones (Caselles-Osorio and García 2007). On the other hand, the continuous feeding mode enhances the removal of sulfate compared to the intermittent ones as reported by Wu et al. (2015).

The impact of intermittent feeding mode and different durations of dry time on vertical-flow constructed wetland treatment efficiency was investigated by Jia et al. (2010). The authors’ results stated that compared to the continuous feeding system, the intermittent one showed lower chemical oxygen demand and total phosphorous removal efficiencies with high ammonium reduction (≥ 90%) due to the high oxygen available in the system during the intermittent feeding operation. This agrees with the results obtained from Fan et al. (2012, 2013), who studied the influence of continuous and intermittent feeding operation on nitrogen removal of free water surface flow and subsurface flow treatment wetlands. Authors’ results showed that in subsurface flow treatment wetlands, the intermittent feeding operation significantly improved ammonium removal, while no significant impact was observed in the free water surface constructed wetland system.

### Impact of environmental factors on constructed wetland behavior

#### Wastewater pH

The pH of wastewater is an important factor that may affect the performance of wetlands, mainly in terms of nitrogen and organic matter removal. For example, substantial alkalinity consumption during the nitrification process leads to a significant drop in pH values of the system, subsequently affecting denitrification rates as discussed by Kadlec and Knight (1996). However, the optimum pH value for the denitrification process can range between 6 and 8, while the highest rate occurs at a pH value of 7.0 to 7.5, as reported by Saeed and Sun (2012). Moreover, Vymazal (2007) noted that a slower rate of denitrification process can occur at a pH value of 5, while an insignificant denitrification rate can be observed at pH values less than 4.

The wastewater pH values are also important for anaerobic degradation processes of organic matter (Saeed and Sun 2012). This is because of the high sensitivity of bacteria responsible for the formation of methane gas in the system. Bacteria can only survive at pH values between 6.5 and 7.5. As a result, the anaerobic degradation process will not complete, if the pH value is not in this range, which leads to volatile fatty acid accumulation in the system and a subsequent drop in the pH value killing all methanogens available in the wetland system as reported by Cooper et al. (1996) and Vymazal (1999).

Considering the reuse of the constructed wetland effluent for irrigation, the treated wastewater pH values are very important. For example, if the pH is very low, the irrigated soil will be acidic resulting in an uptake of all nutrients and elements available in the soil affect negatively plant growth and productivity, while for high water pH values, the media will be basic in nature, which will prevent crops from taking up the necessary elements from the soil, resulting in growth stunting with very low productivity as reported by Almuktar et al. (2017). Based on that, the standard for irrigation water indicated the range of irrigation water pH to be between 6 and 8 (Table 2).

#### Temperature

Several studies have been undertaken to monitor the impact of temperature on wetland treatment processes (Zhang et al. 2014). For example, Trang et al. (2010) studied the wetland behavior in tropical conditions. They found out that there is a significant (*p* < 0.05) impact of higher operation temperature on improving the treatment process in less time, mainly associated with the rate of organic matter degradation, nitrification, and denitrification processes. According to Demin and Dudeney (2003) and Katayon et al. (2008), a high rate of nitrification process can be achieved at a temperature range between 16.5 and 20 °C, while very slow rates occur at temperatures of 5 to 6 °C and above 40 °C as reported by Hammer and Knight (1994), Werker et al. (2002), and Xie et al. (2003). However, the ammonification process will occur optimally at a temperature range of 40 to 60 °C (Vymazal 2007). Moreover, Tunçsiper (2009) reported that ammonia-nitrogen and nitrate-nitrogen removal efficiencies for a constructed wetland were 7 and 9%, respectively, greater in summer than in winter. This is because of the direct link between microbial activity and temperature in the wetlands and the subsequent impact on pollutant removal efficiency, which will generally decline at low temperature due to the reduction in microbial activities (Zhang et al. 2014).

In Shanghai, a study was undertaken to investigate the impact of seasonal temperature on the performance of constructed wetlands (Song et al. 2009). The authors’ results indicated that the treatment efficiency clearly depended on temperature. For example, they found that the removal efficiency of chemical oxygen demand was higher in summer and spring (66.3 and 65.4%, respectively) compared to winter and autumn (59.4 and 61.1% in that order). Also, they discovered that the removal efficiency of ammonia-nitrogen and total phosphorous was higher in summer (54.4 and 35.0%, respectively) than in winter (32.4 and 28.9%, correspondingly). On the other hand, Li et al. (2008) did not indicate substantial differences in chemical oxygen demand removal efficiency at different seasons, while a noticeable difference in removal of nutrients was recorded in summer compared to winter. However, the adverse impact of low temperature on nitrogen and organic matter elimination in constructed wetlands was confirmed by Ruan et al. (2006), Akratos and Tsihrintzis (2007), Zhang et al. (2011), and Zhao et al. (2011).

The wetland treatment efficiency in tropical regions is higher than in temperate regions due to differences in the temperature promoting better plant growth leading to higher up-taking by macrophytes (Kivaisi 2001; Diemont 2006; Katsenovich et al. 2009; Bodin 2013). Moreover, high temperature will increase the microbial activity and subsequently elevate removal processes. For example, the removal efficiency of organic matter will increase at high temperature as the rate of aerobic and anaerobic degradation will increase as well.

On the other hand, high temperature will increase the ammonification rate and plant litter breakdown releasing ammonia-nitrogen and phosphorous from the tropical wetland sediment. As a result, the concentrations of these nutrients in the effluent will be higher than in the influent, which results in negative removal efficiencies in these wetlands.

#### Availability of oxygen

In subsurface flow constructed wetlands, the availability of oxygen is an important environmental factor, which has a direct impact on the treatment performance of the system as it controls nitrification and aerobic degradation of organic matter (Saeed and Sun 2012). However, in horizontal subsurface flow constructed wetlands, which have a saturated substrate (constantly water-logged), there is insufficient oxygen availability leading to inhibition of nitrification processes (Cerezo et al. 2001; Ramirez et al. 2005), while in vertical-flow treatment wetlands, the intermittent feeding mode of wastewater and unsaturated substrate will enhance air diffusion and subsequently increase the availability of oxygen in the system as discussed by Sun et al. (1998) and Noorvee et al. (2007), and this will result in promoting aerobic degradation and nitrification of organic substances (Saeed and Sun 2012).

However, denitrification and anaerobic degradation of organic matter is promoted in horizontal-flow treatment wetlands despite the lack of oxygen availability (Rousseau et al. 2004), indicating the effectivity of these systems in nitrate-nitrogen and organic matter treatment (Saeed and Sun 2012). On the other hand, the rate of oxygen transfer in vertical-flow constructed wetlands is approximately 28 g O_2_/m^2^ day (Cooper 2005), but can be increased by forced aeration leading to improved nitrification processes as reported by Saeed and Sun (2012).

Moreover, Ong et al. (2010) studied the impact of available oxygen on wetland treatment efficiency by comparing the results obtained from two vertical-flow constructed wetlands, one aerated by forced aeration and the other non-aerated. The results showed that the aerated system had higher nitrogen and chemical oxygen demand removals (90 and 94%, respectively) compared to those from the non-aerated system (59 and 90% in this order), indicating a significant impact of forced aeration on nitrogen removal efficiency, but not on organic matter.

These results were confirmed by Stefanakis and Tsihrintzis (2012), who observed high efficiency of organic and nitrogen removal in their wetland systems due to improving system bed aeration. Enhancing aeration of the wetland substrate contributes strongly to the removal of petroleum hydrocarbons in wastewaters, with an efficiency of very closely to 100%, as reported by Wallace et al. (2011). Regarding vertical-flow constructed wetlands, as wastewaters are applied intermittently, then drained vertically from the system by gravity, this will provide the wetland media with a high amount of oxygen supporting aerobic biodegradation processes of organic matter (Vymazal 2007; Stefanakis and Tsihrintzis 2012; Fan et al. 2013; Zhi et al. 2015).

### Application of wetlands in agriculture

Because of the value of wetlands in treating wastewater, several studies have been undertaken to assess the recycling of wetland effluent for different purposes, mainly for agricultural reuse. For example, Cui et al. (2003) studied the treatment of septic tank effluent applying vertical-flow treatment wetlands in China. The author’s results indicated removal efficiencies of 60, 80, 74, 49, and 79% for chemical oxygen demand, biochemical oxygen demand, suspended solids, total nitrogen, and total phosphorus, respectively. Moreover, the total coliform removal rate was between 85 and 96%. The effluent of their experiment was recycled for romaine lettuce and water spinach cultivation. The authors reported that reusing of treated effluent resulted in elevated nitrate levels in the cultivated vegetables. Another study was carried out by Lopez et al. (2006) to investigate the potential for recycling of urban wastewater treated by constructed wetlands in agriculture. Findings indicated removal efficiencies of 85, 65, 75, 42, and 32% for suspended solids, biochemical oxygen demand, chemical oxygen demand, total nitrogen, and total phosphorus, respectively.

Morari and Giardini (2009) assessed pilot-scale vertical-flow constructed wetlands for treating domestic wastewater and subsequent recycling for irrigation purposes. The study results showed that the values for some parameters, which were sufficiently removed from wastewater, complied with the Italian irrigation reuse guidelines, while others, which were poorly removed such as suspended solids and total phosphorus, were restricting the reuse of the treated wastewater. Moreover, Cirelli et al. (2012) showed findings of a recycling scenario, where tertiary-treated municipal wastewater using a constructed wetland was supplied for irrigation of vegetables in Italy. Too high *E. coli* counts in the irrigation water were observed.

Marecos do Monte and Albuquerque (2010) carried out a study of a 21-month monitoring campaign of a horizontal subsurface flow constructed wetland located in rural Portugal. The authors indicated that the low removal efficiency was due to fluctuations of hydraulic loading rate that influenced the hydraulic retention time and the applied loads. Nevertheless, the effluent conductivity, biochemical oxygen demand, chemical oxygen demand, total nitrogen, total phosphorus, potassium, calcium, magnesium, and phytotoxic elements (sodium, chloride, and bromide) were suitable for irrigation reuse according to different international standards, although it is necessary to improve the removal of phosphorous and a final disinfection must be implemented to decrease pathogens. The use of reclaimed water from constructed wetland systems may represent an important water source for irrigation reuse in rural areas of Portugal subjected to water shortages, with important environmental and economic benefits.

According to Vymazal (2014), the basic investment costs for constructed wetlands include land, site investigation, system design, earthwork, liners, filtration (HF and VF CWs) or rooting (FWS CWs) media, vegetation, hydraulic control structures, and miscellaneous costs (e.g., fencing and access roads). However, the proportions of individual costs vary widely in different parts of the world. Also, larger systems demonstrate greater economies for scale. For example, Vymazal and Kröpfelová (2008) summarized available data from horizontal-flow constructed wetlands in the USA, Czech Republic, Portugal, Spain, and Portugal, and found out that excavation costs varied between 7.0 and 27.4% of the total capital cost, while gravel varied between 27 and 53%, liner (13–33%), plants (2–12%), plumbing (6–12%), control structures (3.1–5.7%), and miscellaneous (1.8–12.0%). The total investment costs vary even more, and the cost could be as low as 29 USD per m^2^ in India or 33 USD per m^2^ in Costa Rica, or as high as 257 EUR per m^2^ in Belgium (Vymazal 2011a, b).

In general, the capital costs for subsurface flow constructed wetlands are about the same as for conventional treatment systems. The capital costs for free-water surface-flow constructed wetlands are usually less than for subsurface flow systems, because the costs for media are limited to rooting soil on the bottom of the beds. Constructed wetlands have very low operation and maintenance costs, including pumping energy (if necessary), compliance monitoring, maintenance of access roads and berms, pre-treatment maintenance (including regular cleaning of screens and emptying of septic or Imhoff tanks as well as grit chambers), vegetation harvesting (if applicable), and equipment replacement and repairs. The basic costs are much lower than those for competing concrete and steel technologies by a factor of 2–10 (Vymazal 2005, 2014).

### Potential impact of wastewater irrigation reuse

There are several advantages associated with wastewater recycling for irrigation including the supply of nutrients and trace minerals to plants, potentially leading to higher yields and a decrease in the demand for inorganic fertilizers (Almuktar et al. 2017). However, irrigation with wastewater can also be associated with numerous disadvantages such as potential impacts on public health, crops, soil, and groundwater resources; property values; and ecological and social impacts. Pathogenic microorganisms and heavy metals are among the main challenges affecting human health when irrigating with wastewater. For example, bacteria, viruses, and human parasites such as helminth eggs and protozoa are of particular interest as they are difficult to remove from wastewater and have a substantial impact on human health. These pathogens are responsible for many infectious diseases in both developing and developed countries (Almuktar and Scholz 2016a).

Chemical pollutants available in the wastewater, mainly industrial wastewater, should be taken into consideration when irrigating plants as they will accumulate in plant tissue and then enter the food chain by human consumption. Impacts on soil are of specific importance since they may reduce soil quality in terms of productivity, fertility, and yield. Soil should remain at a good level of chemical and physical characteristics to enable long-term sustainable use and profitable agriculture.

The commonly expected soil problems associated with wastewater use for irrigation are salinization, increased alkalinity, and reduced soil permeability; accumulation of nutrients and potential toxic elements; and microbes in soil irrigated with wastewater (FAO 2003). Another considerable impact associated with wastewater long-term application is the quality of groundwater due to the leaching of salts and nutrients from wastewater below the root zone of plants. However, this impact may depend on several factors such as water table depth, and groundwater quality as well as the drainage of the soil. For example, the impact of leaching nitrate will be determined from the groundwater quality, and in the case of brackish groundwater, leaching nitrate will be of less concern as the water will be invaluable for use. Based on this, the evaluation of groundwater to protect it from the possibility of contamination should be undertaken before application of an irrigation program involving wastewater (FAO 2003; WWAP 2012; 2014; 2015). Since the wetland systems were reported to remove most of the above contaminants adequately (Online Resource 1), the use of reclaimed water from wetland systems may represent an important water source for irrigation reuse (Almuktar and Scholz 2016a).

## Guidelines for decision-making in constructing wetlands for reuse of treated wastewater

Generally, wetland systems are efficient in treating various types of wastewater. However, the effluent quality mainly depends on the influent properties. Based on previous studies, the authors pointed out the following tips to obtain best results when using wetlands for wastewater treatment and subsequent reuse:

- Vertical-flow constructed wetlands perform well in terms of nitrification and poor in denitrification. This is why they are recommended for inflow wastewater that is high in ammonia-nitrogen concentrations.
- Horizontal-flow constructed wetlands perform well in terms of denitrification and poor in nitrification. Consequently, this type is recommended for inflow wastewater of elevated nitrate-nitrogen values.
- Hybrid constructed wetlands are recommended to obtain effluent of low nitrogen levels in terms of ammonia and nitrate.
- Fine wetland substrate is good in removing inflow particles. However, this may result in clogging challenges within the system. Therefore, graduated substrate sizes are recommended for best results in terms of system behavior and effluent quality.
- A long hydraulic detention time will support the treatment process providing more contact time between contaminants and active biomass leading to an improved effluent quality.
- Moderate resting time of wetlands will provide the system with more oxygen supporting the microorganism in the improvement of the effluent properties.
- The inflow loading rate of the wetland can affect directly the effluent quality. A high inflow loading rate may positively affect the treatment process due to nutrients being provided to the microorganisms in the treatment process. Moreover, the effluent may exceed the water quality thresholds. Therefore, dilution of the influent wastewater is recommended.
- Using suitable macrophyte species will affect the treatment efficiency of the wetlands. Choosing proper plant types based on the inflow quality and plant tolerance to the inflow contaminant levels such as nutrients and heavy metals as well as plants tolerance to the salinity is essential.
- The wetland system depth should not be very high as previous studies showed that shallow systems had better results than deeper ones. However, the depth should match the plant root depth to insure best treatment across the whole system depth by plant roots.
- The location of the constructed wetland system will affect the type of wetland to be used. For example, free water surface-flow wetlands are not recommended in urban areas due to the high potential of exposure of pathogens to humans.
- Environmental conditions should be considered when constructing wetlands. For example, at high temperature, evapotranspiration will increase effluent salinity. In such a case, subsurface flow constructed wetlands are highly recommended. However, a high temperature may positively affect the system behavior due to the high activity of system microorganisms resulting in high wastewater treatment efficiency.

## Concluding remarks

This review highlighted the global water scarcity challenge indicating that around half of the world population is likely to experience water stress by 2030 and an increase in the global water demand was estimated to be 55% by 2050. This is due to human population growth, industrial and agricultural activity expansion, global warming, and climate changes contributing to the water scarcity problems in many regions worldwide. Therefore, this review assessed non-conventional water resources to address the increased rates of demand for freshwater. Recycling of wastewater is a widely available alternative option to overcome the shortage in water supply. Insufficient provision of sanitation and wastewater disposal facilities is likely to lead to both environmental and public health challenges. Because of this, wastewater treatment and recycling methods will be vital to provide sufficient freshwater in the coming decades. Since more than 70% of water in the world are consumed for irrigation purposes, the application of treated wastewater for agricultural irrigation has great potential, especially when incorporating the reuse of nutrients like nitrogen and phosphorous, which are important for plant production. Among the current technologies applied in urban wastewater reuse for irrigation, wetland technology was seen as having a great potential in terms of pollutant removal and has advantages in terms of low maintenance costs and required energy.

Constructed wetlands can be classified according to water level, macrophyte, and water movement management. Wastewater characteristics decide upon the wetland class (design) to be used for treatment. Wetland behavior and efficiency concerning wastewater treatment is mainly linked to macrophytes, substrate, hydrology, surface loading rate, influent feeding mode, microorganism availability, and temperature. Having reviewed wastewater reclamation using wetland technology, the following has been concluded:

- Pollution is removed through the process, which is common in natural wetlands, while in constructed wetlands, these processes are undertaken under more controlled circumstances.
- All types of constructed wetlands are very effective in removing organics and suspended solids, whereas removal of nitrogen is lower, but could be improved by using a combination of various types of constructed wetlands.
- Removal of phosphorus is usually low, unless special media with high sorption capacity are used.
- Successful pathogen removal from constructed wetland effluent was challenging.
- Storage of the wetland effluent in lagoons proved beneficial for pathogen removal.
- Using hybrid wetland systems enhances pathogen removal from the effluent as single-stage wetlands cannot meet the standards for irrigation reuse.
- Constructed wetlands require very low or zero energy input, and therefore, operation and maintenance costs are much lower compared to conventional treatment systems.
- In addition to treatment, constructed wetlands are often designed as dual- or multi-purpose ecosystems, which may provide other ecosystem services such as flood control, carbon sequestration, or wildlife habitat.

## Recommendations

Agricultural field parameters and farming practice should be taken into account for an adequate integrated evaluation of the potential to recycle treated wastewater using wetlands in the agricultural sector. The authors strongly recommend the treatment of various wastewater types with wetland technology and subsequent recycling of the corresponding effluents for irrigation purposes. Testing of different crop species is recommended to better understand the growth characteristics and tolerance of individual crops for treating specific wastewaters. Moreover, long-term studies when recycling treated wastewater for irrigation of crops are recommended to assess the potential drawbacks of mineral and microbial contamination on both crops and soils. Definition of guidelines for water reclamation for agricultural reuse requires an integrated assessment from both agricultural practice and wastewater treatment responsible entities.

New knowledge is required concerning the use of urban reclaimed water for irrigation. This knowledge concerns the presence of many micro-pollutants, which are commonly known as compounds of emerging concern in treated wastewaters that can pose risks for the environment and humans when applied for irrigation purposes. More research on, for example, pharmaceuticals and their up-take by crops irrigated with reclaimed water is recommended. These crops are used for human consumption and/or animal forage, and could potentially be harmful. Compounds of emerging concern can also be stress factors for crops irrigated with reclaimed water, and should also be assessed together with conventional water quality parameters such as organic strength, nutrients, and solids in the future.

## Electronic supplementary material

ESM 1 (PDF 311 kb)

ESM 2 (PDF 273 kb)

ESM 3 (PDF 275 kb)

ESM 4 (PDF 288 kb)

ESM 5 (PDF 267 kb)
