# Supplementary material for: Wetlands for wastewater treatment and subsequent recycling of treated effluent: a review
Source: Environ Sci Pollut Res Int. 2018 Jun 29;25(24):23595–623. doi: 10.1007/s11356-018-2629-3 (PMC6096557; doi:10.1007/s11356-018-2629-3)
Supplement: Supplementary file 1 — (PDF 311 kb) [file 11356_2018_2629_MOESM1_ESM.pdf]

Online Resource 1

Wetlands for wastewater treatment and subsequent recycling of treated effluent for irrigation purposes in agriculture: a review

Reviews in Environmental Science and Bio/Technology

Suhad A.A.A.N. Almuktar • Suhail N. Abed • Miklas Scholz

Civil Engineering Research Group, School of Computing, Science and Engineering, The University of Salford, Newton Building, Salford M5 4WT, England, United Kingdom.

Division of Water Resources Engineering, Department of Building and Environmental Technology, Faculty of Engineering, Lund University, P.O. Box 118, 221 00 Lund, Sweden

E-mail address: miklas.scholz@tvrl.lth.se (M. Scholz).

Department of Civil Engineering Science, School of Civil Engineering and the Built Environment, University of Johannesburg, Kingsway Campus, PO Box 524, Aukland Park 2006, Johannesburg, South Africa

Table S1

Wastewater treatment technologies for irrigation purposes (abilities, advantages and disadvantages).

Technology	Ability: Salinity	Ability: Pathogen	Ability: Nutrient and heavy metal	Advantage	Disadvantage
Oxidant					
Sodium hypochlorite (NaOCl)	No electrical conductivity removal (Norton-Brandão et al. 2013)	High bactericidal action (Bixio and Wintgens 2006)	Nitrate removal (NO_3) of 10% and ortho-phosphate- (PO_4) removal of 18% (Üstün et al. 2011)	Low operating costs (Bixio and Wintgens 2006)	High operability; high formation of by products; moderate investment costs (Bixio and Wintgens 2006)
Ozone	-	High bactericidal action (Bixio and Wintgens 2006)	-	Low formation of by-products (Bixio and Wintgens 2006)	High operability; moderate operating costs; high investment costs (Bixio and Wintgens 2006)
Ultraviolet treatment	-	High bactericidal action (Bixio and Wintgens 2006)	-	Low formation of by-products; low operating costs (Bixio and Wintgens 2006)	High operability; moderate investment costs (Bixio and Wintgens 2006)
Photo catalysis with TiO_2	-	High inactivation of coliforms (Rojas-Higuera et al. 2010)	-	Likely use of renewable energy in the case of solar photo catalysis; no formation of by-products; use of inexpensive catalysts and facilities (Lydakis-Simantiris et al. 2010)	Lack of residual bactericidal action; slow kinetic behaviour (Lydakis-Simantiris et al. 2010)

Table S1 (cont.)

Technology	Ability			Advantage	Disadvantage
	Salinity	Pathogen	Nutrient and heavy metal		
Constructed wetland and pond	No removal of electrical conductivity (Pedrero et al. 2010)	Bacterial removal between 1 and 6 log units (Feigin et al. 2012)	Removal in the range of 55% for chromium (Cr) (Arroyo et al. 2010); between 25% and 35% for nickel (Ni), between 25% and 87% for zinc (Zn) and 9% for copper (Cu) (Galletti et al. 2010); 33% for cadmium (Cd) and 75% for cobalt (Co) (Pedrero et al. 2010)	Low maintenance costs and energy usage; no formation of by-products (Brissaud 2007; Ghermandi et al. 2007)	Large footprint; efficiency depending on meteorological conditions (Brissaud 2007; Ghermandi et al. 2007)
Medium filtration	-	Faecal coliform removal between 0.6 and 1.5 log units (Li et al. 2008)	Achievement of final concentration of 5 mg/l of total nitrogen (TN) and 4-10 mg/l of ortho-phosphate-phosphorous (PO_4-P) (Metcalf 2003)	Low investment costs; low operating costs (Li et al. 2008)	Low removal of faecal coliform (Li et al. 2008)

Table S1 (cont.)

Technology	Ability			Advantage	Disadvantage
	Salinity	Pathogen	Nutrient and heavy metal		
Membrane filtration	Removal or preservation of nutrients according to the pore size; reverse osmosis (RO) reduced 90% of electrical conductivity (Jacob et al. 2010); Nano-filtration (NF) rejects only divalent cations allowing most monovalent ions, which include nutrients, to pass and hardly alters the salinity (Chang et al. 2005)	Bacterial removal higher than 5 log units (Lazarova et al. 1999)	Removal of 83% of sodium (Na) and 80% of chlorine (Cl) (Oron et al. 2008); removal of nutrients, sodium ions and divalent cations (Chang et al. 2005); removals in the range of 75% for chromium (Cr) and > 80% for arsenic (As) (Fatone 2005)	Simultaneous disinfection and removal of electrical conductivity (Norton-Brandão et al. 2013)	High investment costs; high operational costs (Lazarova et al. 1999)
Electrolysis	-	Effective disinfection with low current charges (Rodrigo et al. 2010)	-	Effective in killing a wide spectrum of microorganisms (Drogui et al. 2001)	Formation of significant amounts of perchlorates (Bergmann et al. 2009)

References

Arroyo P, Ansola G, de Luis E (2010) Effectiveness of a full-scale constructed wetland for the removal of metals from domestic wastewater. Wat Air Soil Pollut 210:473–481.doi: http://dx.doi.org/10.1007/s11270-009-0272-9

Bergmann MEH, Rollin J, Iourtchouk T (2009) The occurrence of perchlorate during drinking water electrolysis using BDD anodes. Electrochimica Acta 54:2102–2107. doi: http://dx.doi.org/10.1016/j.electacta.2008.09.040

Bixio D, Wintgens T (2006) Water Reuse System Management Manual. AQUAREC, Office for Official Publications of the European Communities, Luxembourg.

Brissaud F (2007) Low technology systems for wastewater treatment: perspectives. Wat Sci Technol 5:1–9. doi: http://dx.doi.org/10.2166/wst.2007.120

Chang I, Lee E, Oh S, Kim Y (2005) Comparison of SAR (sodium adsorption ratio) between RO and NF processes for the reclamation of secondary effluent. Wat Sci Technol 51:313–318.

Drogui P, Elmaleh S, Rumeau M et al (2001) Oxidising and disinfecting by hydrogen peroxide produced in a two-electrode cell. Wat Res 35:3235–3241. doi: http://dx.doi.org/10.1016/S0043-1354(01)00021-5

Fatone F, Bolzonella D, Battistoni P et al (2005) Removal of nutrients and micropollutants treating low loaded wastewaters in a membrane bioreactor operating the automatic alternate-cycles process. Desalination 183:395–405. doi: http://dx.doi.org/10.1016/j.desal.2005.02.055

Feigin A, Ravina I, Shalhevet J (2012) Irrigation with treated sewage effluent: management for environmental protection (Vol. 17). Springer-Verlag, Berlin.

Galletti A, Verlicchi P., Ranieri E (2010) Removal and accumulation of Cu, Ni and Zn in horizontal subsurface flow constructed wetlands: Contribution of vegetation and filling medium. Sci Tot Environm 408:5097–5105. doi: http://dx.doi.org/10.1016/j.scitotenv.2010.07.045

Ghermandi A, Bixio D, Traverso P et al (2007) The removal of pathogens in surface-flow constructed wetlands and its implications for water reuse. Wat Sci Technol 56:207-216. doi: http://dx.doi.org/10.2166/wst.2007.511

Jacob M, Guigui C, Cabassud C, Darras H, Lavison G, Moulin L (2010) Performances of RO and NF processes for wastewater reuse: Tertiary treatment after a conventional activated sludge or a membrane bioreactor. Desalination 250:833–839. doi: http://dx.doi.org/10.1016/j.desal.2008.11.052

Lazarova V, Savoye P, Janex ML, et al. (1999) Advanced wastewater disinfection technologies: State of the art and perspectives. Wat Sci Technol 40:203–213. doi: http://dx.doi.org/10.1016/S0273-1223(99)00502-8

Li L, Li Y, Biswas DK et al (2008) Potential of constructed wetlands in treating the eutrophic water: evidence from Taihu Lake of China. Biores Technol 99:1656–1663.

Lydakis-Simantiris N, Riga D, Katsivela E et al (2010) Disinfection of spring water and secondary treated municipal wastewater by TiO_2. Desalination 250:351–355. doi: http://dx.doi.org/10.1016/j.desal.2009.09.055

Metcalf E (2003) Wastewater Engineering, Treatment and Reuse. McGraw-Hill, New York.

Norton-Brandão D, Scherrenberg SM, Van Lier JB (2013) Reclamation of used urban waters for irrigation purposes – a review of treatment technologies. J Environm Managem 122:85–98. doi: http://dx.doi.org/10.1016/j.jenvman.2013.03.012

Oron G, Gillerman L, Buriakovsky N et al (2008) Membrane technology for advanced wastewater reclamation for sustainable agriculture production. Desalination 218:170–180. doi: http://dx.doi.org/10.1016/j.desal.2006.09.033

Pedrero F, Kalavrouziotis I, Alarcón JJ et al (2010) Use of treated municipal wastewater in irrigated agriculture — review of some practices in Spain and Greece. Agricult Wat Managem 97:1233–1241. doi: http://dx.doi.org/10.1016/j.agwat.2010.03.003

Rodrigo MA, Cañizares P, Buitrón C et al (2010) Electrochemical technologies for the regeneration of urban wastewaters. Electrochimica Acta 55:8160–8164. doi: http://dx.doi.org/10.1016/j.electacta.2010.01.053

Rojas-Higuera N, Sánchez-Garibello A, Matiz-Villamil A et al (2010) Evaluation of three methods for the inactivation of coliforms and Escherichia coli present in domestic wastewaters used in irrigation. Universitas Scientiarum 15:139–149.

Üstün GE, Solmaz SKA, Çiner F et al (2011) Tertiary treatment of a secondary effluent by the coupling of coagulation–flocculation–disinfection for irrigation reuse. Desalination 277:207–212. doi: http://dx.doi.org/10.1016/j.desal.2011.04.032
