# Supplementary material for: Wetlands for wastewater treatment and subsequent recycling of treated effluent: a review
Source: Environ Sci Pollut Res Int. 2018 Jun 29;25(24):23595–623. doi: 10.1007/s11356-018-2629-3 (PMC6096557; doi:10.1007/s11356-018-2629-3)
Supplement: Supplementary file 2 — (PDF 273 kb) [file 11356_2018_2629_MOESM2_ESM.pdf]

Online Resource 2

Wetlands for wastewater treatment and subsequent recycling of treated effluent: a review

Reviews in Environmental Science and Bio/Technology

Suhad A.A.A.N. Almuktar • Suhail N. Abed • Miklas Scholz

Civil Engineering Research Group, School of Computing, Science and Engineering, The University of Salford, Newton Building, Salford M5 4WT, England, United Kingdom.

Division of Water Resources Engineering, Department of Building and Environmental Technology, Faculty of Engineering, Lund University, P.O. Box 118, 221 00 Lund, Sweden

E-mail address: miklas.scholz@tvrl.lth.se (M. Scholz).

Department of Civil Engineering Science, School of Civil Engineering and the Built Environment, University of Johannesburg, Kingsway Campus, PO Box 524, Aukland Park 2006, Johannesburg, South Africa

Constructed wetland background details

In developed countries, there are initiatives to control micro-pollutants in wastewater, while in developing countries, plant managers struggle to control macro-pollutants, which can, however, be successfully controlled by constructed wetlands (Mustafa 2013). Constructed wetlands treat wastewater at considerably lower cost compared to traditional systems, and allow for the reuse of effluent in the agricultural sector (Greenway 2005). According to Belmont et al. (2004) and Wang et al. (2005), treatment of urban wastewater using wetland technology has been reported to be suitable for irrigation of plants. Moreover, constructed wetland systems show high efficiencies in removing most contaminants in domestic wastewater including chemicals (organics, heavy metals and trace elements, etc.) and microorganisms (bacteria, viruses, parasites, etc.) as reported by Kivaisi (2001) and Gross et al. (2007).

References

Belmont MA, Cantellano E, Thompson S et al (2004) Treatment of domestic wastewater in a pilot-scale natural treatment system in central Mexico. Ecol Eng 23:299–311.

Greenway M (2005) The role of constructed wetlands in secondary effluent treatment and water reuse in subtropical and arid Australia. Ecol Eng 25:501–509. doi: http://dx.doi.org/10.1016/j.ecoleng.2005.07.008

Gross A, Shmueli O, Ronen Z et al (2007) Recycled vertical flow constructed wetland (RVFCW) — a novel method of recycling greywater for irrigation in small communities and households. Chemosphere 66:916–923. doi: http://dx.doi.org/10.1016/j.chemosphere.2006.06.006

Kivaisi AK (2001) The potential for constructed wetlands for wastewater treatment and reuse in developing countries: a review. Ecol Eng 16:545–560. doi: http://dx.doi.org/10.1016/S0925-8574(00)00113-0

Mustafa A (2013) Constructed wetland for wastewater treatment and reuse: a case study of developing country. Int J Environm Sci Developm 4:20–24.

Wang X, Bai X, Qiu J et al (2005) Municipal wastewater treatment with pond-constructed wetland system: a case study. Wat Sci Technol 51:325–329.
