# Supplementary material for: Wetlands for wastewater treatment and subsequent recycling of treated effluent: a review
Source: Environ Sci Pollut Res Int. 2018 Jun 29;25(24):23595–623. doi: 10.1007/s11356-018-2629-3 (PMC6096557; doi:10.1007/s11356-018-2629-3)
Supplement: Supplementary file 3 — (PDF 275 kb) [file 11356_2018_2629_MOESM3_ESM.pdf]

Online Resource 3

Wetlands for wastewater treatment and subsequent recycling of treated effluent: a review

Reviews in Environmental Science and Bio/Technology

Suhad A.A.A.N. Almuktar • Suhail N. Abed • Miklas Scholz

Civil Engineering Research Group, School of Computing, Science and Engineering, The University of Salford, Newton Building, Salford M5 4WT, England, United Kingdom.

Division of Water Resources Engineering, Department of Building and Environmental Technology, Faculty of Engineering, Lund University, P.O. Box 118, 221 00 Lund, Sweden

E-mail address: miklas.scholz@tvrl.lth.se (M. Scholz).

Department of Civil Engineering Science, School of Civil Engineering and the Built Environment, University of Johannesburg, Kingsway Campus, PO Box 524, Aukland Park 2006, Johannesburg, South Africa

Details on constructed wetland substrate

Under saturation conditions, the pores in the wetland substrate will be filling-up with water instead of air. In this case, the dissolved oxygen available in the water will be consumed by microbes. Since oxygen will be more than that restored during the circulation phase, the media will become anoxic. Moreover, the substrate will be anaerobic under inundation conditions (Scholz 2006, 2010; Stefanakis et al. 2014). Using a sand and gravel mixture in the wetland substrate is recommended, as this can improve the system behaviour in terms of hydraulic conductivity and contaminant removal (Stottmeister et al. 2003). Moreover, the use of fine-grained instead of large-grained media in a wetland system is preferable as it will provide better conditions for microorganism growth and subsequently improve pollutant biodegradation (Dordio and Carvalho 2013), but at the same time, the fine aggregate will lead to substrate clogging (Song et al. 2015). Langergraber et al. (2003) suggested using compound layers of gravel arranged by size increment from the top. However, their results showed that clogging in such a system is highly likely. Other studies showed that using an anti-sized reed bed system instead of the traditional mono-sized one, is very effective in terms of pollutant removal from heavily contaminated wastewater (Sun et al. 2007), while Song et al. (2015) indicated that the use of large-size packing media will result in high removal of chemical oxygen demand and ammonia-nitrogen, while reducing the likelihood of system clogging.

References

Dordio AV, Carvalho AJP (2013) Organic xenobiotics removal in constructed wetlands, with emphasis on the importance of the support matrix. J Hazard Mat 252:272–292.

Langergraber G, Haberl R, Laber J et al (2003) Evaluation of substrate clogging processes in vertical flow constructed wetlands. Wat Sci Technol 48:25–34.

Scholz M (2006) Wetlands systems to control urban runoff. Elsevier, Amsterdam.

Scholz M (2010) Wetland systems — storm water management control. SpringerVerlag, Berlin.

Song X, Ding Y, Wang Y et al (2015) Comparative study of nitrogen removal and bio-film clogging for three filter media packing strategies in vertical flow constructed wetlands. Ecol Eng 74:1–7.

Stefanakis A, Akratos CS, Tsihrintzis VA (2014) Vertical flow constructed wetlands: eco-engineering systems for wastewater and sludge treatment. Newnes, Oxford.

Stottmeister U, Wießner A, Kuschk P et al (2003) Effects of plants and microorganisms in constructed wetlands for wastewater treatment. Biotechnol Adv 22:93–117.

Sun G, Zhao YQ, Allen SJ (2007) An alternative arrangement of gravel media in tidal flow reed beds treating pig farm wastewater. Wat Air Soil Pollut 182:13-19.
