# Supplementary material for: Wetlands for wastewater treatment and subsequent recycling of treated effluent: a review
Source: Environ Sci Pollut Res Int. 2018 Jun 29;25(24):23595–623. doi: 10.1007/s11356-018-2629-3 (PMC6096557; doi:10.1007/s11356-018-2629-3)
Supplement: Supplementary file 4 — (PDF 288 kb) [file 11356_2018_2629_MOESM4_ESM.pdf]

Online Resource 4

Wetlands for wastewater treatment and subsequent recycling of treated effluent: a review

Reviews in Environmental Science and Bio/Technology

Suhad A.A.A.N. Almuktar • Suhail N. Abed • Miklas Scholz

Civil Engineering Research Group, School of Computing, Science and Engineering, The University of Salford, Newton Building, Salford M5 4WT, England, United Kingdom.

Division of Water Resources Engineering, Department of Building and Environmental Technology, Faculty of Engineering, Lund University, P.O. Box 118, 221 00 Lund, Sweden

E-mail address: miklas.scholz@tvrl.lth.se (M. Scholz).

Department of Civil Engineering Science, School of Civil Engineering and the Built Environment, University of Johannesburg, Kingsway Campus, PO Box 524, Aukland Park 2006, Johannesburg, South Africa

Table S2
Common substrate types used (in no particular order) in constructed wetland systems with source.

Substrate type		
Natural material	Industrial by-products	Artificial products
Sand (Saeed and Sun 2013)	Slag (Cui et al. 2010)	Activated carbon (Ren et al. 2007)
Gravel (Calheiros et al. 2008; Almuktar and Scholz 2015, 2016 a,b)	Fly ash (Xu et al. 2006)	Light weight aggregates (Saeed and Sun 2012)
Clay (Calheiros et al. 2008)	Coal cinder (Ren et al. 2007)	Compost (Saeed and Sun 2012)
Calcite (Ann et al. 1999)	Alum sludge (Babatunde et al. 2010)	Calcium silicate hydrate (Li et al. 2011)
Marble (Arias et al. 2001)	Hollow brick crumbs (Ren et al. 2007)	Ceramsite (Li et al. 2011)
Vermiculite (Arias et al. 2001)	Moleanos limestone (Mateus et al. 2012)	
Bentonite (Xu et al. 2006)	Wollastonite tailings (Hill et al. 1997)	
Dolomite (Ann et al. 1999)	Oil palm shell (Chong et al. 2013)	
Limestone (Tao and Wang 2009)		
Shell (Seo et al. 2005)		
Shale (Saeed and Sun 2012)		
Peat (Saeed and Sun 2012)		
Wollastonite (Brooks et al. 2000)		
Maerl (Saeed and Sun 2012)		
Zeolite (Bruch et al. 2011)		

References

Almuktar SAAAN, Scholz,M (2015) Microbial contamination of *Capsicum annuum* irrigated with recycled domestic wastewater treated by vertical-flow wetlands. Ecol Eng 82:404–414. doi: http://dx.doi.org/10.1016/j.ecoleng.2015.05.029

Almuktar SAAAN, Scholz M (2016a) Mineral and biological contamination of soil and *Capsicum annuum* irrigated with recycled domestic wastewater. Agricult Wat Managem 167:95–109. doi: http://dx.doi.org/10.1016/j.agwat.2016.01.008

Almuktar SAAAN, Scholz M (2016b) Experimental assessment of recycled diesel spill-contaminated domestic wastewater treated by reed beds for irrigation of Sweet Peppers. Int J Environm Res Publ Health, 13:208. doi: http://dx.doi.org/10.3390/ijerph13020208

Ann Y, Reddy K, Delfino J (1999) Influence of chemical amendments on phosphorus immobilization in soils from a constructed wetland. Ecol Eng 14:157–167.

Arias CA, Del Bubba M, Brix H (2001) Phosphorus removal by sands for use as media in subsurface flow constructed reed beds. Wat Res 35:1159–1168.

Babatunde A, Zhao Y, Zhao X (2010) Alum sludge-based constructed wetland system for enhanced removal of P and OM from wastewater: concept, design and performance analysis. Biores Technol 101:6576–6579.

Calheiros CS, Rangel AO, Castro PM (2008) Evaluation of different substrates to support the growth of *Typha latifolia* in constructed wetlands treating tannery wastewater over long-term operation. Biores Technol 99:6866–6877.

Chong HLH, Chia PS, Ahmad MN (2013) The adsorption of heavy metal by Bornean oil palm shell and its potential application as constructed wetland media. Biores Technol 130:181–186.

Hill DT, Payne VWE, Rogers JW, et al (1997) Ammonia effects on the biomass production of five constructed wetland plant species. Biores Technol 62:109–113.

Li CJ, Wan MH, Dong Y et al (2011) Treating surface water with low nutrients concentration by mixed substrates constructed wetlands. J Environm Sci Health Part A 46:771–776.
Mateus DM, Vaz MM, Pinho HJ (2012) Fragmented limestone wastes as a constructed wetland substrate for phosphorus removal. Ecol Eng 41:65–69.
Ren Y, Zhang B, Liu Z et al (2007) Optimization of four kinds of constructed wetlands substrate combination treating domestic sewage. Wuhan Univ J Nat Sci 12:1136-1142.
Saeed T, Sun G (2013) A lab-scale study of constructed wetlands with sugarcane bagasse and sand media for the treatment of textile wastewater. Biores Technol 128:438–447.
Xu D, Xu J, Wu J et al. (2006) Studies on the phosphorus sorption capacity of substrates used in constructed wetland systems. Chemosphere 63:344–352.
