# Supplementary material for: Wetlands for wastewater treatment and subsequent recycling of treated effluent: a review
Source: Environ Sci Pollut Res Int. 2018 Jun 29;25(24):23595–623. doi: 10.1007/s11356-018-2629-3 (PMC6096557; doi:10.1007/s11356-018-2629-3)
Supplement: Supplementary file 5 — (PDF 267 kb) [file 11356_2018_2629_MOESM5_ESM.pdf]

Online Resource 5

Wetlands for wastewater treatment and subsequent recycling of treated effluent: a review

Reviews in Environmental Science and Bio/Technology

Suhad A.A.A.N. Almuktar • Suhail N. Abed • Miklas Scholz

Civil Engineering Research Group, School of Computing, Science and Engineering, The University of Salford, Newton Building, Salford M5 4WT, England, United Kingdom.

Division of Water Resources Engineering, Department of Building and Environmental Technology, Faculty of Engineering, Lund University, P.O. Box 118, 221 00 Lund, Sweden

E-mail address: miklas.scholz@tvrl.lth.se (M. Scholz).

Department of Civil Engineering Science, School of Civil Engineering and the Built Environment, University of Johannesburg, Kingsway Campus, PO Box 524, Aukland Park 2006, Johannesburg, South Africa

Details on constructed wetland microorganisms

Most fungi utilise saprophytic nutrition, which mainly depends on the degradation of dead organic matter. Fungi are plentiful in wetland systems and play a significant role in water treatment. Moreover, fungi are environmentally vital in wetlands, because they recycle a substantial proportion of carbon and nutrients.

Furthermore, fungi can symbiotically live with algae and higher plants, increasing their ability for nutrient sorption from air, water and soil. However, the presence of fungi in wetland systems containing toxic metals and chemicals will limit the production of algae and higher plants as the recycling of nutrients in such systems will be condensed. In the wetland ecosystem, fungi grow naturally on dead plant litter layers (Kadlec and Wallace 2008). However, the interaction between microorganisms, substrate and plants of a wetland system can directly affect the pollutant removal ability of a constructed wetland system (Scholz 2010). For example, plants in the wetland system consist of two parts, above- and below-ground biomass, providing a large surface area for microbial growth (Brix 1997). Moreover, as wetland macrophytes grow and subsequently die, the decaying leaves and stems produces multi-layers of litter (organic debris). This will create a porous substrate layer providing a substantial area for microbial attachment, which will directly benefit the function of water quality improvement (Brix 1997). Moreover, the wetland plants will transfer oxygen through their hollow tissue and release it from the roots to the rhizosphere, supporting aerobic degradation of organic matter and the nitrification process as discussed by Brix (1997).

References

Brix H (1997) Do macrophytes play a role in constructed treatment wetlands? Wat Sci Technol 35:11–17.
Kadlec RH, Wallace S (2008) Treatment Wetlands. 2nd ed., CRC press, Boca Raton, FL.
Scholz M (2010) Wetland systems — storm water management control. SpringerVerlag, Berlin.
